# Dietary polysaccharides in the management of inflammatory bowel disease: recent advances

**DOI:** 10.3389/fnut.2026.1779617

**Published:** 2026-05-11

**Authors:** Siwei Yang, Jie Wu, Junjie Xiang, Mengmeng Wang, Pingping Shen, Lingyan He

**Affiliations:** 1 School of medicine, Shaoxing University, Shaoxing, Zhejiang, China; 2 Department of Traditional Chinese Medicine, Shaoxing People's Hospital, Shaoxing, Zhejiang, China; 3 Department of Pharmacy, Qingdao Eighth People's Hospital, Qingdao, Shandong, China; 4 Department of Gastroenterology, Yinzhou District Second Hospital, Ningbo, Zhejiang, China

**Keywords:** diet, gut microbiota, immunity, inflammatory bowel disease, polysaccharides

## Abstract

The increasing global burden of inflammatory bowel disease (IBD) has renewed awareness of the limitations and adverse effects of conventional pharmacotherapies, highlighting the need for safe, naturally derived, and mechanistically precise interventions. This review summarizes current understanding of IBD pathogenesis and the biological activities of dietary polysaccharides, with particular emphasis on their diverse protective functions in the gut. Robust preclinical evidence indicates that dietary polysaccharides can markedly alleviate colitis through multiple, interconnected mechanisms. These include reshaping the gut microbial ecosystem and its metabolites—such as short-chain fatty acids, tryptophan-derived indoles, and bile acids—restoring both the mechanical and chemical components of the intestinal barrier, and remodeling cytokine networks while rebalancing key immune cell subsets, including Th17/Treg and M1/M2 macrophages. In parallel, dietary polysaccharides modulate critical inflammatory signaling pathways, notably nuclear factor kappa-B (NF-κB), mitogen-activated protein kinase (MAPK), and NOD-like receptor protein 3 (NLRP3) inflammasome, thereby suppressing excessive intestinal inflammatory activity. Despite these promising experimental findings, clinical evidence remains limited, and important questions regarding structure–activity relationships, *in vivo* metabolic fate, and long-term safety and efficacy in patients with IBD have yet to be fully addressed. Future research should integrate emerging technologies such as nanotechnology and artificial intelligence to dissect molecular mechanisms in greater depth and to guide the rational design of polysaccharide-based therapeutics, dietary supplements, and functional foods tailored to individual patient profiles, thereby advancing precision nutrition strategies for IBD management.

## 1 Introduction

Inflammatory bowel disease (IBD) refers to a group of chronic intestinal disorders characterized by persistent inflammation and immune dysregulation. The two major clinical subtypes are ulcerative colitis (UC) and Crohn's disease (CD). CD may involve any segment of the gastrointestinal tract from the mouth to the anus and affects all layers of the intestinal wall. Typical symptoms include abdominal pain, fever, bowel obstruction, and

bloody or mucous diarrhea (1). In contrast, UC is confined to the colonic mucosa and is defined by continuous superficial ulceration, usually presenting with bloody diarrhea, mucus discharge, and tenesmus (2). IBD follows a relapsing-remitting clinical course. Without effective disease control, persistent inflammation can give rise to severe complications such as intra-abdominal abscess, fistula formation, intestinal strictures, and obstruction. Long-standing disease also markedly increases the risk of gastrointestinal malignancies, with substantial impacts on quality of life and long-term prognosis, as well as significant economic burden (3). Historically, IBD was most prevalent in industrialized and high-income regions, with the greatest disease burden observed in North America and Europe (4). However, recent epidemiological data indicate a striking shift in global distribution, with rapidly rising incidence in newly industrialized countries across Asia, South America, and the Middle East. In China, the prevalence of IBD has increased more than thirtyfold since the late 1950s, transforming from a rare clinical entity to a common digestive disorder (5).

Current therapeutic strategies for IBD rely primarily on aminosalicylates such as 5-aminosalicylic acid and sulfasalazine. For moderate to severe disease or insufficient response to first-line therapy, oral corticosteroids, immunomodulators including thiopurines, methotrexate, and Janus kinase inhibitors, as well as biologic agents such as tumor necrosis factor inhibitors and interleukin (IL) 12/23 inhibitors, are commonly employed (6). Despite their clinical utility, these treatments have notable limitations including substantial inter-patient variability in therapeutic response and considerable adverse effects with long-term use. For instance, sulfasalazine may cause nausea, dyspepsia, headache, vomiting, and abdominal discomfort (7), while thiopurines are associated with pancreatitis and bone marrow suppression (8). In addition, primary or secondary loss of response is frequently observed, and the high cost of biologics restricts their availability in developing countries.

These challenges highlight the urgent need for more effective, safer, and cost-efficient anti-inflammatory therapies. Thus, dietary polysaccharides represent a particularly promising category. These naturally occurring macromolecules are abundant in commonly consumed foods such as *Astragalus* (9), bitter melon (10), goji berries (11), and yam (12), and exhibit high safety profiles while avoiding the toxicities associated with synthetic drugs. They are inexpensive, widely available, and possess diverse pharmacological properties including antioxidant activity, free radical scavenging, anti-inflammatory effects, and the ability to maintain intestinal homeostasis (13). In recent years, dietary polysaccharides have shown substantial potential in the prevention and treatment of IBD. Numerous studies have demonstrated that they alleviate intestinal inflammation by restoring gut microbial balance, reducing inflammatory cell infiltration and mucosal injury, and suppressing the release of pro-inflammatory cytokines such as tumor necrosis factor α (TNF-α) and IL-6 (14–17).

## 2 Pathogenesis of IBD

The pathogenesis of IBD is complex and not yet fully understood, although substantial evidence indicates that genetic predisposition, environmental exposures, gut microbiota imbalance, and immune dysregulation all play critical roles (18).

### 2.1 Genetic susceptibility and environmental factors

Family and twin studies have long suggested a heritable component in IBD development (19). The prevalence of CD or UC among first-degree relatives of affected individuals is significantly higher than the background rate of approximately 2 to 15 percent, with genetic influence being more pronounced in CD than in UC (20). In addition to genetic factors, epidemiological, clinical, and experimental data support strong associations between IBD and a wide range of environmental exposures. These include smoking, dietary patterns, medication use, geographic and socioeconomic factors, psychological stress, microbial agents, intestinal permeability, and prior appendectomy, among others (21).

### 2.2 Gut microbiota dysbiosis and intestinal barrier dysfunction

The composition of the gut microbiota is pivotal to the development of IBD. In a healthy microbiome, the gastrointestinal tract is dominated by three major bacterial phyla: Bacteroidetes, Firmicutes, and Proteobacteria. Microbial communities also vary markedly along the length of the gut, with Veillonellaceae, Pseudomonadaceae, and Streptococcaceae being more prevalent in the upper gastrointestinal tract, whereas Lachnospiraceae, Bacteroidaceae, and Ruminococcaceae predominate in the lower intestine (22). These microorganisms contribute to intestinal homeostasis by supporting metabolic functions, shaping immune responses, and limiting pathogen colonization (23).

In experimental models, Paik and colleagues showed that fecal microbiota from IBD-prone SPF *Smad3*^−/−^ mice induced more severe intestinal inflammation in germ-free *Smad3*^−/−^ recipients than microbiota from *Smad3*^−/−^ mice without IBD (24). Consistent with these findings, patients with IBD typically exhibit reduced microbial diversity, decreased abundances of beneficial taxa within Firmicutes and Bacteroidetes, and an expansion of Gammaproteobacteria (25).

Dysbiosis can promote intestinal barrier dysfunction through intertwined metabolic and inflammatory mechanisms. Loss of beneficial, short-chain fatty acid–producing bacteria such as *Faecalibacterium prausnitzii* reduces butyrate availability, which is associated with diminished expression of tight junction proteins including Zonula Occludens (ZO)-1 and occludin and consequent weakening of the epithelial mechanical barrier (26). Meanwhile, overgrowth of Proteobacteria and other pathobionts increases exposure to lipopolysaccharide (LPS) and other microbial products, activating epithelial inflammatory signaling, promoting goblet cell loss, and thinning the mucus layer via reduced MUC2, thereby compromising the chemical barrier that normally separates luminal microbes from the epithelium (27). As permeability increases, luminal antigens and LPS gain access to the circulation, amplifying both local mucosal inflammation through cytokines such as TNF-α and IL-1β and systemic inflammatory responses. This inflammatory amplification further damages barrier integrity, creating a self-reinforcing cycle that sustains chronic intestinal inflammation (28).

### 2.3 Immune dysregulation

Immune dysregulation in IBD involves imbalances within both innate and adaptive immune responses, accompanied by aberrant activation of immune cells, cytokines, and signaling pathways that collectively disrupt the equilibrium between pro-inflammatory and anti-inflammatory mechanisms (29). The innate immune system comprises elements such as the mucus layer, epithelial barrier, macrophages, monocytes, neutrophils, and natural killer cells, and is capable of initiating rapid, non-specific responses (30). When pathogen-associated molecular patterns are recognized by pattern recognition receptors (PRRs) such as Toll-like receptors (TLRs) and NOD-like receptors (NLRs), downstream signaling cascades are triggered, resulting in the production of pro-inflammatory cytokines, chemokines, and antimicrobial peptides. These events promote phagocytosis, antigen presentation, and ultimately activation of the adaptive immune system, leading to inflammatory responses (31).

Adaptive immunity relies on the antigen-specific actions of B and T cells and therefore operates more slowly than innate immunity. In IBD, T helper (Th) cell responses become dysregulated, with exaggerated activation of Th1 and Th17 cells driving increased production of pro-inflammatory cytokines including TNF-α, IFN-γ, IL-1, IL-6, IL-12, and IL-23. At the same time, regulatory T cells (Tregs) are reduced in number or exhibit impaired function, limiting their ability to restrain effector T-cell activation. This breakdown of immune tolerance aggravates intestinal inflammation and contributes to disease progression (32, 33).

## 3 Overview of dietary polysaccharides

Polysaccharides, also known as glycans, are polymeric carbohydrates formed by the linkage of multiple monosaccharides through glycosidic bonds generated by dehydration condensation. With a general formula of (C_6_H_10_O_5_)_*n*_, they constitute an essential component of dietary fiber. Based on monosaccharide composition, polysaccharides can be classified into homopolysaccharides composed of a single type of monosaccharide and heteropolysaccharides containing two or more types of monosaccharide units (34). Dietary polysaccharides are widely distributed in nature and can be categorized into plant polysaccharides such as pectin and beta-glucans, fungal polysaccharides such as lentinan and ganoderan, animal polysaccharides such as chitosan and hyaluronic acid, and microbial polysaccharides such as xanthan gum and gellan gum. Among these, plants serve as one of the richest natural sources.

The biological functions of polysaccharides arise from their complex structural features. Their primary structures include monosaccharide composition and ratios, types and sequences of glycosidic linkages, molecular weight, and its distribution (35). Different dietary polysaccharides possess distinct monosaccharide profiles. For example, apple polysaccharides contain rhamnose, galactose, arabinose, glucose, and galacturonic acid in specific proportions (36), while *Poria cocos* polysaccharides consist predominantly of mannose, glucosamine hydrochloride, glucose, galactose, and fucose in defined molar ratios (37). The nature and arrangement of glycosidic linkages, such as alpha or beta linkages and 1 → 4, 1 → 6, or 1 → 3 bonds, determine the rigidity and chemical stability of the polysaccharide backbone. For instance, the alpha 1,4 glucan backbone is fundamental to the anti-inflammatory and barrier-protective activities of *Astragalus* polysaccharides (9). Molecular weight and its dispersity strongly influence solubility, viscosity, and bioavailability (38). Polysaccharides within an appropriate molecular weight range often display enhanced biological activities; low-molecular-weight Tremella polysaccharides, for example, demonstrate superior immunomodulatory and antioxidant effects compared with their high-molecular-weight counterparts (39).

Beyond primary structure, the three-dimensional conformation of polysaccharides plays a decisive role in shaping their functional properties. The triple-helix structure is a notable example and serves as a key determinant of the immunomodulatory activity of fungal beta-glucans. This structural motif promotes macrophage proliferation and stimulates the release of immune mediators such as nitric oxide, TNF-α, and IL-1β (40), while also enhancing swelling capacity and water and oil retention (41). Even small variations in structural parameters or chemical modifications, including acetylation, sulfation, and carboxymethylation, can markedly alter or strengthen their biological activities (42). Carboxymethylated derivatives of blackcurrant polysaccharides, for instance, exhibit improved antioxidant properties, including enhanced scavenging of hydroxyl and superoxide radicals, greater protection against lipid peroxidation, and reduced erythrocyte hemolysis (43). Phosphorylated onion polysaccharides demonstrate antioxidant activity comparable to that of vitamin C (44).

Over the past decade, dietary polysaccharides isolated from foods have drawn increasing attention due to their diverse pharmacological properties, including antitumor, hepatoprotective, antioxidant, anti-obesity, antidiabetic, and neuroprotective activities, as well as gut microbiota-modulating effects—properties that underpin their wide-ranging applications in nutrition and pharmacology (45, 46). Lentinan, a soluble dietary fiber derived from *Lentinula edodes*, exhibits multiple biological activities and is especially well known for its anti-tumor potential. It inhibits tumor progression by inducing apoptosis, suppressing proliferation, and blocking key oncogenic pathways such as Janus kinase (JAK) 2/signal transducer and activator of transcription 3 (STAT3) and phosphoinositide 3-kinase/protein kinase B (Akt). When combined with nanomaterials, its tumor-targeting capacity is further enhanced, and it acts synergistically with chemotherapeutic agents including cisplatin, gemcitabine, and docetaxel to improve efficacy and reduce toxicity (46). *Poria cocos* polysaccharides exhibit hepatoprotective, antioxidant, and anti-nephritic properties (47). *Momordica charantia* polysaccharides have been reported to exert anti-obesity and anti-diabetic activities and to modulate gut microbiota composition (10). Recent studies also suggest that certain polysaccharides exert neuroprotective effects by suppressing neuroinflammation, preserving the gut–brain barrier, and regulating neurotransmitter balance (48). In the context of intestinal health, dietary polysaccharides such as *Codonopsis pilosula* polysaccharides (CPPS) (15), oat beta-glucans (49), and *Hericium erinaceus* polysaccharides (50) possess complex structures that resist degradation in the upper gastrointestinal tract and can reach the colon intact, where they serve as fermentable substrates for gut microbiota. This inherent colon-targeting characteristic provides a natural delivery advantage and forms an important mechanistic basis for their potential in IBD intervention.

## 4 Mechanisms of dietary polysaccharides in IBD

### 4.1 Modulation of gut microbiota balance: microbiota–host interactions

**Figure 1:**
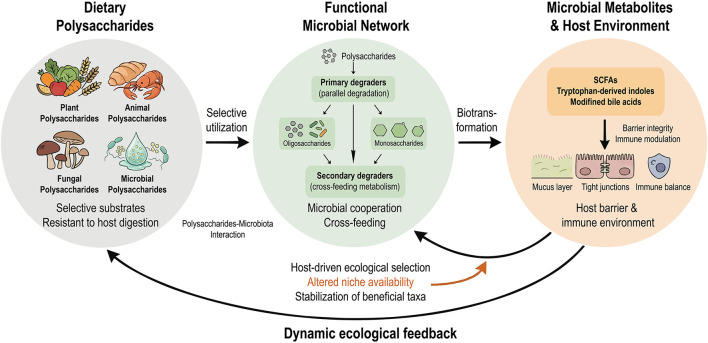
Bidirectional interaction between dietary polysaccharides and gut microbiota in IBD. Dietary polysaccharides derived from plant, animal, fungal, and microbial sources reach the colon as selective substrates resistant to host digestion. These polysaccharides are utilized by gut microbiota and undergo parallel degradation by primary degraders, generating diverse intermediate products such as oligosaccharides and monosaccharides. These intermediates are further metabolized through microbial cross-feeding networks involving secondary degraders, leading to the production of bioactive metabolites, including short-chain fatty acids (SCFAs), tryptophan-derived indoles, and modified bile acids. These metabolites contribute to the maintenance of intestinal barrier integrity and immune homeostasis. In turn, the altered host microenvironment exerts ecological selection on the gut microbiota, reshaping community composition and stabilizing beneficial taxa, thereby forming a dynamic feedback loop between dietary polysaccharides, microbiota, and host responses.

The human gastrointestinal tract harbors a diverse and highly mutualistic microbial community. Dynamic equilibrium of this gut microbiota is fundamental to intestinal health, and interactions between the microbiota and the host are central to the maintenance of this balance. Prebiotics are defined as substrates that are selectively utilized by host microorganisms conferring a health benefit (51). Such substances are not confined to dietary supplements but are also widely present in natural foods such as whole grains and vegetables, where they help preserve gastrointestinal homeostasis, modulate microbial composition by promoting the growth of beneficial bacteria, and inhibit the expansion of potentially pathogenic species (52). Dietary polysaccharides are typical natural prebiotics and act as important mediators in initiating microbiota–host regulatory processes. Targeted modulation of the microbial community by these polysaccharides and restoration of ecological balance represent key steps in improving intestinal disease state. Figure 1 provides a schematic overview of how dietary polysaccharides interact with functional microbial networks and subsequently influence barrier integrity and immune homeostasis in IBD. Table 1 summarizes key dietary polysaccharides and their microbiota-modulating effects, providing a systematic overview of the evidence discussed below.

**Table 1:** Representative dietary polysaccharides that reshape gut microbiota composition and microbial metabolites in experimental colitis/IBD models.

Polysaccharides	Dietary source	Experimental models	Chemical/molecular features	Increased taxa	Decreased taxa	Key metabolites/functional changes	References
*Gastrodia elata* polysaccharides (GEP/GBP)	*Gastrodia elata*	- *In vitro*: simulated saliva–gastric–intestinal digestion + human fecal fermentation model - *In vivo*: DSS-induced IBD mouse model	- Mw: Digestion products (GEP-I): 41.328 kDa and 1.801 kDa; purified fractions GBP1 (1.435 × 106 Da) and GBP2 (1.913 × 106 Da). - Monosaccharides: glucose (90.56%−98.95%) with galactose, galacturonic acid, rhamnose, arabinose, and glucuronic acid. - Structures: α-1,4-glucan, α-1,4,6-glucan, β-1,6-glucan.	- *Genera: Bifidobacterium, Collinsella, Prevotella, Faecalibacterium, Roseburia, Ligilactobacillus, Alloprevotella*	- Phyla: Verrucomicrobiota; - *Genera: Shigella, Dorea, Desulfovibrio, Blautia, Akkermansia, Bacteroides*, Escherich/Shigella	- Metabolites: ↑ acetate, propionate, butyrate, caproate; ↓ gut pH; - Pathways: ↑ tryptophan metabolism, vitamin B6 metabolism, steroid hormone biosynthesis; - Inflammation: IL-1β, TNF-α, IL-6	(52, 53)
*Lycium barbarum* polysaccharides (LBP)	*Lycium barbarum*	- *In vitro* probiotic culture (e.g., *Lactobacillus acidophilus, Bifidobacterium longum*); - *In vivo*: Kunming mice, gavage for 14 days	- Monosaccharides: glucose (molar proportion 6.52); arabinose:rhamnose:xylose:mannose:galactose = 0.18:0.81:0.07:2.17:0.23 (molar ratio), total ≈ 10	- Phyla: Firmicutes, Proteobacteria; - Family: Prevotellaceae; - Genera*:* *Lactobacillus acidophilus, Akkermansia, Lactobacillus*	- Phyla: Bacteroidetes	- Metabolites: ↑ TGF-β, IL-6, sIgA; - Inflammation: ↑ thymus index and spleen index	(11)
*Codonopsis pilosula* polysaccharides (CPPS)	*Codonopsis pilosula*	- *In vivo*: DSS-induced UC mouse model; FMT validation model	- Mn 6.056 kDa; Mw 29.386 kDa; PDI 4.852; - Monosaccharides: rhamnose 3.82%, arabinose 24.67%, galactose 10.11%, glucose 10.31%, galacturonic acid 51.10%	- Phyla: Firmicutes/Bacteroidetes (F/B) ratio; Verrucomicrobia; - Genera: *Ligilactobacillus, Akkermansia, Faecalibaculum, Odoribacter*	- Phyla: Bacteroidetes, Proteobacteria; - Genera: *Duncaniella, Turicibacter, Escherichia*	- Metabolites: ↑ acetate and butyrate; activates GPR43/GPR109A; - Inflammation: ↓ NLRP3 inflammasome activation; ↓ IL-1β, IL-18, IL-6, TNF-α	(15)
Oat β-glucan (BG)	Oat	- *In vivo*: DSS-induced UC mouse model	- Mw 614.543 kDa (homogeneous polysaccharide). - Monosaccharides: glucose 97.47 mol% (β-linked glucan). - Structure: linear β-glucan with β-(1 → 3) and β-(1 → 4) glycosidic bonds	- Phylum: Bacteroidetes	- Phyla: Firmicutes, Proteobacteria	- Metabolites: ↑ acetate, propionate, butyrate; - Inflammation: ↓ TNF-α, IFN-γ, IL-6; - Intestinal barrier: ↑ ZO-1, occludin, claudin-1	(48)
Sea buckthorn polysaccharides (SBP)	Sea buckthorn	- *In vivo*: DSS-induced UC mouse model; FMT validation model	- Mw 58.78 kDa. - Monosaccharides (molar ratio): fructose:rhamnose:arabinose:galactose:glucose:xylose:galacturonic acid = 0.65:5.97:53.43:6.05:6.50:2.59:24.82	- Families: Lactobacillaceae; Lachnospiraceae; Enterobacteriaceae; - Genera: *Ligilactobacillus, Lactobacillus, Bacteroides, Akkermansia, Lactobacillus acidophilus, Bifidobacterium longum*	- Families: Lactobacillaceae; Christensenellaceae; - Genera: *Dubosiella, Escherich/Shigella*, Lachnospiraceae_NK4A136_group; uncultured *Lactobacillus* sp.	- Metabolites: ↑ SCFAs (acetate, propionate, butyrate, caproate, valerate, etc.) and bile acids including CA and DCA; - Inflammation: improves Th17/Treg balance; inhibits IκB/NF-κB; ↑ FFAR2/FFAR3	(46, 102)
Turmeric polysaccharides (TPS)	Turmeric	- *In vivo*: DSS-induced UC mouse model	- Monosaccharides (molar ratio): ribose:xylose:fructose:glucose:galactose = 5.8:0.8:19.3:49.4:24.7.	- Phylum: Bacteroidetes; - Genera: *Lactobacillus, Akkermansia, Bifidobacterium, Clostridia-UCG-014*	- Phylum: Firmicutes	- Metabolites: ↑ IAA, IAAId, SCFAs (acetate, propionate, butyrate, valerate); ↓ tryptophan and I3AM; - Intestinal barrier: ↑ ZO-1 and occludin; - Inflammation/oxidation: activates AhR and ↑ IL-22; ↓ TNF-α, IL-17, IL-10; ↓ CD45^+^ immune cells and F4/80^+^ macrophage infiltration; ↑ GSH/GSSG ratio	(58)
Tea polysaccharides (TPS)	Tea leaves	- *In vivo*: DSS-induced UC mouse model	- Monosaccharides: Composition included rhamnose, arabinose, galactose, glucose, ribose, and galacturonic acid (molar ratio 2.15:6.64:10.29:6.70:2.41:20.74).	- Phylum: Bacteroidetes; - Family: Lactobacillaceae; - Genus: *Lactobacillus*	- Phylum: Proteobacteria - Family: Enterobacteriaceae; Peptostreptococcaceae	- Intestinal barrier: ↑ ZO-1 and Claudin-1; - Therapeutic effect: ↓ disease activity index (DAI)	(65)
*Ganoderma lucidum* polysaccharides (GLP)	*Ganoderma lucidum*	- *In vitro*: LPS-induced RAW264.7/HT-29/NCM460 inflammation models; DSS-induced NCM460 model; - *In vivo*: AOM/DSS-induced CAC and DSS-induced acute/chronic colitis mouse models	- Mw 25.0 kDa; - Monosaccharides: glucose 78.15%, mannose 15.69%, etc.	- Genera: *Lactobacillus, Bifidobacterium, Bacteroides*, Lachnospiraceae_NK4A136_group	- Family: Enterobacteriaceae; - Genera: *Desulfovibrio, Oscillibacter, Escherichia–Shigella, Akkermansia*	- Metabolites: ↑ propionate and butyrate; activates GPR43; - Inflammatory pathways: inhibits TLR4/MyD88/NF-κB and MAPK; - Barrier/Immune regulation: ↑ ZO-1, occludin, and MUC2; modulates Th17/Treg; ↓ IL-17 and RORγT; ↑ FOXP3 and IL-10	(64, 87)
Quinoa polysaccharides (QPS)	Quinoa	- *In vivo*: LPS-induced systemic acute inflammation mouse model	- Mw: Q1 : 4.283 × 104 Da and Q2: 4.88 × 103 Da; - Monosaccharides: Q1: arabinose:glucose:galacturonic acid = 16.97:11.69:12.03; Q2: arabinose:glucose = 82.69:17.31; - Structure: polysaccharide content 91.75%	- Phylum: Bacteroidota; - Genera: *Bacteroides, Lactobacillus, Roseburia, Alloprevotella, Odoribacter, and Lachnospiraceae_NK4A136_group*	- Phyla: Firmicutes, Proteobacteria, Patescibacteria; - Genera: *Desulfovibrio, Enterococcus*	- Metabolites: ↑ SCFAs (acetate, propionate, butyrate, isovalerate, etc.); ↑ xanthine and hypoxanthine - Immune regulation: ↓ IL-6, TNF-α, IL-1β; ↑IL-10	(94)
Hawthorn polysaccharides (HAW1–2)	Hawthorn	- *In vitro*: LPS-induced IEC-6 cell model; - *In vivo*: DSS-induced IBD mouse model	- Mw 8.94 kDa; - Monosaccharides: include arabinose, galactose, glucose (molar ratio not specified); - Structures: heteropolysaccharide	- Genera: *Bacteroides, Alistipes, Odoribacter, Dubosiella*	- Family: Coriobacteriaceae_UCG-002; - Genus: *Turicibacter*	- Metabolites: ↑ acetate, propionate, butyrate; Alkali-soluble purple sweet potato polysaccharide (ASPP)	Purple sweet potato	- *In vivo*: DSS-induced colitis mouse model	- Mw 180 kDa; - Monosaccharides (molar ratio): glucose:mannose:rhamnose:arabinose:xylose = 53.3:7.6:2.8:1.9:1.0; - Structures: backbone 1,4-α-D-glucan with branching at O-6	- Genera: Lachnospiraceae_NK4A136_group, *Lactobacillus, Roseburia, Bifidobacterium*	- Genera: *Bacteroides, Staphylococcus, Parasutterella, Desulfovibrio*	- Metabolites: ↑ acetate and propionate; - Gut microbita: restores Firmicutes/Bacteroidetes ratio	(95)
*Cistanche deserticola* polysaccharides (CDPS)	*Cistanche deserticola*	- *In vivo*: DSS-induced IBD mouse model	- Mw 3.31–1019.07 kDa; - Monosaccharides: glucose 63.27%, arabinose 15.56%, galactose 9.10%	- Phylum: Firmicutes; Family: Lachnospiraceae_NK4A136_group	- Phyla: *Campylobacterota*, Desulfobacterota; - Genera: *Helicobacter, Bacteroides*	- Inflammatory pathways: inhibits SRC/EGFR/PI3K/AKT; - Immune regulation: ↑ IL-10	(97)
Rattan pepper polysaccharides (RPP)	Rattan pepper	- *In vivo*: DSS-induced IBD mouse model	- Monosaccharides (molar ratio): arabinose:galactose:glucose:galacturonic acid:rhamnose = 30.46:34.50:15.49:10.40:5.07, etc.	- Family Coriobacteriaceae_UCG-002; - Genera: - Lachnospiraceae_NK4A136_group; *Desulfovibrio*; *Enterorhabdus*;-Species: *Olsenella_phocaeensis*; *Streptococcus_danieliae*	- Genera: Parasutterella; Olsenella; Parabacteroides; Alistipes	- Metabolites: ↑ acetic acid, propionic acid, butyric acid, and other SCFAs and bile acid metabolism; - Inflammatory pathways: inhibits TLR4/NF-κB; activates CREB/BDNF	(103)
Pumpkin polysaccharides (PPs)	Pumpkin	- *In vitro*: LPS/Nigericin-induced THP-1 inflammation model; - *In vivo*: DSS-induced UC mouse model	- Mw 31 kDa; Monosaccharides (molar ratio): mannose:rhamnose:galacturonic acid:galactosamine:glucose:xylose = 1.58:3.51:34.54:1.00:3.25:3.02; - Structures: homogeneous polysaccharide with α-1,4-galacturonic acid backbone	- Phylum: Campylobacterota; - Genera: *Helicobacter*; Lachnospiraceae NK4A136 group; *Mailhella*; *Mucispirillum*	- Phyla: Bacteroidetes; Deferribacteres; Proteobacteria; - Genera: Bacteroides; Culturomica; Mucispirillum; Escherichia-Shigella; Alistipes	- Metabolites: ↑ 5-HIAA; - Inflammatory pathways: ↑ PPARγ nuclear translocation; ↓ MAPK/NF-κB; ↓ TNF-α, IFN-γ and IL-1β; ↑ IL-4 and IL-10 - Gut microbita: restores Firmicutes/Bacteroidetes ratio	(16)
Glycyrrhiza polysaccharides (GP)	Licorice	- *In vivo*: CTX-induced immunosuppression + intestinal mucosal injury mouse model; FMT validation model	- Mw 6.5 kDa; - Structures: contains 1,6-linked glucose residues; low-molecular-weight heteropolysaccharide	- Phylum: Bacteroidota; - Genera: *Alistipes*; *Bacteroides*; *Ligilactobacillus*; Muribaculaceae_unclassified; Lachnospiraceae_NK4A136_group; Clostridia_vadinBB60_group; *Colidextribacter*	- Phyla: Firmicutes; Proteobacteria; - Genera: Clostridiales_unclassified; *Candidatus Arthromitus*; Firmicutes_unclassified; *Clostridium*	- Metabolites: ↑ acetate and propionate; - Inflammatory pathways: ↑ IFN-γ, IL-2, IL-4, IgM, IgG and SIgA; maintains Th1/Th2 balance; ↑ CD4^+^/CD8^+^ T cells and IgM/IgG/sIgA	(107)
*Hericium erinaceus* polysaccharides (HECP)	*Hericium erinaceus*	- *In vivo*: DSS-induced UC mouse model	- Mw 86.67 kDa; - Monosaccharides: homogeneous polysaccharide containing glucan components	- Phylum: Firmicutes; Order: Clostridiales; Species: Akkermansia muciniphila; - Description: beneficial commensal microbiota (reversing DSS-induced dysbiosis)	- Phyla: Verrucomicrobia; Actinobacteria; Bacteroidetes; - Genera: Desulfovibrio; Arthrobacter spp.; Methylibium sp.; Succinivibrio sp.	- Inflammatory pathways: ↓ phosphorylation of NF-κB, PI3K/Akt, and MAPK pathways - Oxidative stress and inflammatory mediators: ↓ NO, MDA and MPO; ↑ total SOD activity; - Cytokines and downstream effectors: ↓ IL-1β, IL-6, and TNF-α; ↓ COX-2 and iNOS	(49)
Safflower polysaccharides (SPS)	Safflower	- *In vivo*: DSS-induced acute UC mouse model	- Monosaccharides: arabinose, glucose, galactose, inositol acetate	- Phyla: Firmicutes; Verrucomicrobiota; - Genera: Akkermansia; Limosilactobacillus; Bifidobacterium; unidentified Lachnospiraceae	- Phylum: Bacteroidota; - Genera: *Bacteroides*; *Desulfovibrio*	- Inflammation: inhibits STAT3/NF-κB; ↓↓IL-1β, IL-6, IL-8, TNF-α, IL-17, IL-20, IFN-γ, CHI3L1 ↑IL-10/IL-22; - Intestinal barrier: ↑ ZO-1, occludin and claudin-1; ↑ mucins (Muc1/Muc2) and goblet cell protection; ↓ intestinal permeability (↓ serum FITC–dextran)	(80)
*Poria cocos* polysaccharides (PCP)	*Poria cocos*	- *In vitro*: validation with Wnt/β-catenin inhibitor FH535; - *In vivo*: SPF C57BL/6 mice	- Mw 11.583 kDa; Mw/Mn = 1.19; - Monosaccharides: mannose:D-glucosamine HCl:glucose:galactose:fucose = 15.308:0.967:28.723:31.631:23.371; - Structures: backbone contains t-Gal(p), 6-Gal(p), and 2,6-Gal(p)	- Phylum: Bacteroidota; - Family: Muribaculaceae; - Genus: *Bacteroides*	- Phylum: Firmicutes	- Metabolites: ↑ SCFAs (acetate, propionate, isobutyrate, isovalerate, etc.); - Intestinal barrier: ↑ ZO-1 and occludin; ↑ MUC2/β-defensin/sIgA; ↑ Wnt/Lrp5/β-catenin; - Immune regulation: ↑ IL-2, IL-4, IL-6, IL-10, TGF-β and IFN-γ	(83)
*Polygonatum cyrtonema* polysaccharide (PCYP)	*Polygonatum cyrtonema*	- *In vitro*: Caco-2/RAW264.7 co-culture model; - *In vivo*: DSS-induced UC mouse model; antibiotic-depleted UC mouse model	- Mw 5.65 kDa; - - Monosaccharides (molar ratio): fructose:glucose = 28:1	- Genera: *Lactobacillus*; *Akkermansia*; *Bacteroides*; *Blautia*	- Phylum: Proteobacteria; - Genera: *Helicobacter; Escherichia–Shigella; Desulfovibrio;*- Species: *Ruminococcus torques* group	- Intestinal barrier: ↑ ZO-1 and occludin; ↑ MUC2; ↓ intestinal permeability; - Microbiota-independent effect: directly ↓ IL-6, IL-18, IL-17A, IL-1β, TNF-α, IFN-γ; ↑ IL-10; - Signaling + immune balance: inhibits MAPK (ERK, JNK, p38) and NF-κB activation; restores Th17/Treg balance	(77)

AOM, azoxymethane; BA, bile acid; BAs, bile acids; BG, β-glucan; CA, cholic acid; CD, Crohn's disease; CPPS, *Codonopsis pilosula* polysaccharides; CTX, cyclophosphamide; DAI, disease activity index; DCA, deoxycholic acid; DSS, dextran sulfate sodium; FMT, fecal microbiota transplantation; FXR, farnesoid X receptor; GBP, *Gastrodia elata* polysaccharides; GBP1, fraction 1 of *Gastrodia elata* polysaccharides; GBP2, fraction 2 of *Gastrodia elata* polysaccharides; GEP, *Gastrodia elata* polysaccharides; GEP-I, digested products of GEP; GPR43, G protein-coupled receptor 43; GPR109A, G protein-coupled receptor 109A; IAA, indole-3-acetic acid; IAAId, indole-3-acetaldehyde; I3AM, indole-3-acetamide; IBD, inflammatory bowel disease; IFN-γ, interferon-gamma; IgA, immunoglobulin A; IgG, immunoglobulin G; IgM, immunoglobulin M; IL, interleukin; LBP, *Lycium barbarum* polysaccharides; LPS, lipopolysaccharide; Mn, number-average molecular weight; Mw, weight-average molecular weight; NF-κB, nuclear factor kappa-B; NLRP3, NLR family pyrin domain containing 3; PCYP: *Polygonatum cyrtonema* polysaccharide; PDI, polydispersity index; PXR, pregnane X receptor; SCFAs, short-chain fatty acids; sIgA, secretory immunoglobulin A; SPF, specific pathogen-free; TGF-β, transforming growth factor-β; Th1, T helper 1; Th2, T helper 2; TLR4, Toll-like receptor 4; TNF-α, tumor necrosis factor-α; UC, ulcerative colitis.

#### 4.1.1 Restoration of microbial ecology

A primary feature of this regulatory process is the adjustment of the ratio between beneficial and pathogenic bacteria. Studies have shown that administration of *Gastrodia elata* polysaccharides markedly increases the relative abundance of potentially anti-inflammatory genera such as *Bifidobacterium, Ligilactobacillus*, and *Alloprevotella*. These beneficial microbes not only compete with pathogens for nutrients and ecological niches, thereby limiting their overgrowth, but also secrete antimicrobial substances that directly inhibit colonization by opportunistic pathogens such as *Bacteroides, Shigella*, and *Escherichia* species (53, 54). *Lycium barbarum* polysaccharides (LBP) selectively support the growth of probiotic bacteria. In mice, LBP supplementation increases the abundance of Proteobacteria and Firmicutes while reducing the proportion of Bacteroidetes (11). Recovery of microbial diversity is a crucial indicator of ecological restoration. In IBD, microbial diversity is typically decreased, whereas polysaccharide intervention can reverse the decline in alpha diversity and improve community stability, as reflected by increases in the Shannon index and related metrics. LBP, for instance, promotes the expansion of potentially beneficial taxa such as *Akkermansia, Lactobacillus*, and members of the Prevotellaceae, thereby reshaping the dominant community structure of the gut microbiota (11).

#### 4.1.2 Regulation of microbial metabolites

Microbial metabolites serve as key messengers in microbiota–host interactions, and their regulation constitutes another essential dimension of this mechanism. Compared with healthy individuals, patients with IBD exhibit significantly reduced numbers of bacteria capable of fermenting dietary fiber into short chain fatty acids (SCFAs) in both intestinal mucosa and feces. SCFAs such as acetate, propionate, and butyrate are critical metabolites for intestinal homeostasis (55). They provide an important energy source for colonocytes, strengthen the intestinal barrier, and exert potent immunoregulatory effects, among which the actions of butyrate are particularly prominent. Butyrate activates G protein-coupled receptors (GPRs) on the cell surface, including GPR 41, GPR43, and GPR109A, triggers signaling cascades that modulate immune function, and supports epithelial energy metabolism (56).

Recent work by Jiaxin Zhou and colleagues demonstrated that CPPS have promising therapeutic potential in UC. In a mouse model of experimental colitis induced by 3 percent dextran sulfate sodium (DSS), seven days of CPPS administration significantly increased the production of acetate and butyrate compared with DSS alone. Western blot analysis further suggested that these effects were linked to modulation of the SCFA-GPR-NLRP3 signaling axis (15). Consistent findings have been reported for oat β-glucan, which can be fermented by gut microbiota to selectively enhance SCFA production and thereby ameliorate DSS induced colitis in mice (49). Sea buckthorn polysaccharides similarly increase the abundance of SCFA-producing bacteria and elevate colonic SCFA levels following intervention (57).

Tryptophan, an essential amino acid, can be converted by gut microbes into a variety of metabolites such as indoles and kynurenines. The regulatory mechanisms associated with these metabolites share certain overlap with those of SCFAs but also exhibit unique features (58). Turmeric polysaccharides increase the abundance of tryptophan metabolizing probiotics including *Lactobacillus* and *Clostridium* cluster UCG 014, modulate microbiota dependent tryptophan catabolites such as indole-3-acetic acid, and activate the aryl hydrocarbon receptor pathway in colonic cells. This pathway plays a critical role in immune cell differentiation and maintenance of the intestinal barrier (59).

Bile acids represent another class of key microbial derived metabolites. Secondary bile acids, including lithocholic acid, can activate nuclear receptors such as Takeda G protein-coupled receptor 5 and the farnesoid X receptor (FXR) in the intestine and liver, thereby regulating metabolic homeostasis and influencing the course of IBD (60, 61). The gut microbiota is the central regulator of bile acid metabolism. *Astragalus* polysaccharide fraction G2 (APS-G2), which is rich in linear alpha 1,4 glucans, has been shown to enrich beneficial bacteria such as Bifidobacterium and *Lactobacillus*, optimize secondary bile acid profiles, and alleviate DSS induced colitis through FXR-mediated signaling pathways (9). These findings suggest that dietary polysaccharides may attenuate intestinal inflammation by reshaping microbial communities, optimizing secondary bile acid production, and modulating nuclear receptor pathways including FXR and pregnane X receptor.

#### 4.1.3 Re-establishment of microecological homeostasis

Ultimately, these regulatory actions converge on the reestablishment of microecological homeostasis. On one hand, polysaccharides with adhesive properties can reduce bacterial translocation. Mechanistically, they may directly interfere with the binding of pathogenic bacteria, such as adherent invasive *Escherichia coli* (62), whose surface adhesin FimH binds to intestinal epithelial cells. In parallel, by reshaping the microbiota and reducing the proportion of pathogens, polysaccharides decrease the risk of translocation at its source. These two processes work together to prevent inappropriate migration of gut bacteria. On the other hand, polysaccharide intervention can markedly suppress the release of endotoxin. LPS, a glycolipid component of the outer membrane of Gram negative bacteria, is a major driver of both acute and chronic inflammation. In chronic settings, the excess LPS burden is often not derived from exogenous infection but from increased endogenous production within the gut, which is sustained by the resident microbiota and by LPS present in food (63). Excessive LPS activates Toll like receptor 4 and, through adaptor proteins such as myeloid differentiation primary response protein 88 (MyD88) and TIR domain containing adapters, induces activation of nuclear factor kappa B (NF-κB), leading to robust inflammatory responses (64). *Ganoderma lucidum* polysaccharides (GLP) have been shown to ameliorate azoxymethane/DSS-induced dysbiosis by inhibiting Toll-like receptor 4 (TLR4)-MyD88-NF-κB signaling, increasing SCFA production, and alleviating endotoxemia (65). Tea polysaccharides similarly inhibit the overgrowth of Gram negative bacteria, thereby reducing the source of LPS and attenuating chronic inflammation (66). In this context, dietary polysaccharides act as important regulators of microbiota–host interactions and represent promising interventions to suppress chronic intestinal inflammation.

### 4.2 Restoration of intestinal barrier function: physical and biochemical dual barriers

Disruption of the intestinal barrier architecture can trigger uncontrolled immune responses within the gut microenvironment or permit unrestrained translocation of microbiota, ultimately leading to various diseases including IBD (67). Integrity of the intestinal barrier is therefore a central line of defense against microbial invasion and their metabolites, and coordinated repair of both physical and biochemical barriers is critical for rebuilding this defense system. Dietary polysaccharides, such as soybean polysaccharides (68), GLP (65), and *Astragalus* polysaccharides (9), exert multi-level regulatory effects during this repair process by acting on several structural and functional components of the barrier. The physical and biochemical barriers do not operate in isolation. The former limits penetration of harmful substances through structural integrity, whereas the latter uses chemical defense systems to neutralize threats. Their dynamic cooperation forms a three-dimensional protective network along the intestinal surface. Table 2 summarizes the compositional characteristics and pharmacological information of dietary-derived polysaccharides used for the treatment of IBD through intestinal barrier regulation.

**Table 2:** Representative dietary polysaccharides that restore intestinal barrier function in experimental colitis models: evidence and mechanisms.

Polysaccharides	Dietary source	Experimental models	Chemical/molecular features	Intestinal barrier	Mechanism	References
Oat β-glucan (BG)	Oat grain (*Avena sativa*)	- *In vivo*: DSS-induced UC mouse model	- **Mw** 614.543 kDa (homogeneous); - **Monosaccharides:** 97.47 mol% glucose; - **Structures:** linear β-glucan with β-(1 → 3) and β-(1 → 4) linkages; purification not specified	- **Physical barrier:** TJPs (ZO-1, Occludin, Claudin-1, Claudin-4)	- **Barrier repair:** ↑ TJPs at mRNA/protein levels; - **Microbiome regulation:** remodeled microbiota and ↑ SCFA-related taxa; - **Metabolic regulation:** ↑ acetate, propionate, butyrate and affected sphingolipid & glycerophospholipid metabolism; - **Inflammation:** ↓ TNF-α, IFN-γ, IL-6 and apoptosis/infiltration.	(48)
Turmeric polysaccharides (TPS)	Turmeric	- *In vivo*: DSS-induced UC mouse model	- **Monosaccharides (molar ratio):** ribose:xylose:fructose:glucose:galactose = 5.8:0.8:19.3:49.4:24.7	- **Physical barrier:** TJPs (ZO-1, Occludin)	- **Barrier repair:** ↑ ZO-1 and Occludin; - **Microbiome regulation:** ↑ beneficial genera (e.g., ↑*Lactobacillus*, ↑*Clostridium* UCG-014), ↓ Firmicutes, ↑ diversity; - **Metabolic regulation:** ↑ SCFAs and tryptophan metabolites (IAA, IAAId), ↓ tryptophan and I3AM; - **Inflammation/oxidation:** ↓ TNF-α, IL-10, IL-17 and IL-22; ↑ AhR; ↓ oxidative stress.	(58)
*Tremella aurantialba* polysaccharides (TA2-1)	*T. aurantialba*	- ***In vivo***: DSS-induced UC mouse model with FMT; - ***In vivo***: ferroptosis-induced Caco-2	- **Mw** 127 kDa; - **Monosaccharides (molar ratio):** mannose:xylose:glucuronic acid:glucose:fucose:rhamnose = 59.2:23.2:13.9:1.6:1.7:0.4; - **Structures:** A β-(1 → 3)-mannan backbone, with O-2–substituted side chains containing terminal xylose (T-Xylp), β-(1 → 3)-xylopyranose (1 → 3-Xylp), β-(1 → 4)-glucuronic acid (1 → 4-GlcAp), and terminal mannose (T-Manp)	- **Physical barrier:** TJPs (Claudin-1, ZO-1); - Ferroptosis targets: GPX4, FTH1, ACSL4	- **Barrier repair:** ↑ Claudin-1/ZO-1, ↓ permeability; - **Microbiome regulation:** ↑ beneficial microbes; - **Ferroptosis inhibition**: ↑ GPX4/FTH1, ↓ ACSL4, ↓ lipid peroxidation; - **Inflammation:** ↓ IL-6/TNF-α/MCP1	(71)
Longan pulp polysaccharides (LP; LPIa)	Longan pulp	- *In vitro*: LPS-stimulated Caco-2/RAW264.7 co-culture; - *In vivo*: CTX-induced injury mouse model	- **Mw:** the weight-average molecular weights (Mw) of LPIa, LPIIa, LPIIIa, and LPIVa were 147.0 kDa, 159.3 kDa, 19.4 kDa, and 44.0 kDa, respectively; - **Monosaccharides:** LPIa consisted of rhamnose, ribose, arabinose, xylose, mannose, glucose, and galactose, with a molar ratio of 0.99:1.37:34.61:1.48:1.73:5.86:55.16; LPIIa consisted of rhamnose, ribose, arabinose, xylose, glucose, and galactose, with a molar ratio of 1.05:1.00:22.88:1.01:2.59:34.58; LPIIIa consisted of rhamnose, ribose, fucose, arabinose, mannose, glucose, and galactose, with a molar ratio of 14.46:1.85:2.31:46.17:1.00:1.97:20.99; LPIVa consisted of rhamnose, ribose, arabinose, mannose, glucose, and galactose, with a molar ratio of 4.71:0.38:25.03:1.00:2.53:15.50.	- **Physical barrier:** TJPs (ZO-1, Claudin-1, Claudin-4, Occludin), MUC2, E-cadherin	- **Barrier repair:** ↑ ZO-1, Claudin-1, Claudin-4, Occludin, MUC2, E-cadherin; -**Structural synergy:** the porous structure reinforced junction stability; -**Tissue repair:** ↑ villus regeneration and mucosal repair	(17)
Yam polysaccharides (YP)	Yam	- *In vitro*: IEC-6 epithelial model	Not specified	- **Physical barrier:** TJPs (ZO-1, Occludin, Claudin-1), F-actin	- **-Skeletal adjustment:** ↑ F-actin stability & monolayer integrity; -**pathways:** ↓ ROCK/RhoA signaling; - **Barrier repair:** ↑ TJPs (ZO-1, Occludin, Claudin-1); ↑ TEER; ↓ bacterial translocation	(12)
*Ganoderma lucidum* polysaccharides (GLP)	*Ganoderma lucidum*	- *In vitro*: LPS-induced inflammation (RAW264.7/HT-29/NCM460) - *In vivo*: AOM/DSS-induced CAC mouse model; DSS-induced acute/chronic colitis mouse model	- **Mw** 25.0 kDa; **Monosaccharides:** β-glycosidic bonds; glucose 78.15%, mannose 15.69%; - **Structures:** Water-soluble heteropolysaccharide containing β-glycosidic bonds	- **Physical barrier:** TJPs (ZO-1, Occludin), MUC2, F-actin, goblet cells	- **Barrier repair:** ↑ ZO-1/Occludin/MUC2 & goblet cells; ↓ ulcers/crypt damage; -**Microbiota shift:** ↑ Lactobacillus/Bifidobacterium/Bacteroides, ↓ Escherichia–Shigella; ↑α-diversity; -Metabolic synergy: ↑ SCFAs & ↑ GPR43; -**Immune rebalance:** **↓** IL-17/RORγt, ↑ Foxp3/IL-10; ↓ TLR4/MyD88/NF-κB and ↓ MAPK	(64, 87)
*Polygonatum cyrtonema* polysaccharides (PCYP)	*P. cyrtonema*	- *In vitro*: Caco-2/RAW264.7 co-culture - *In vivo*: DSS-induced UC mouse model; antibiotic-depleted model	- **Mw** 5.65 kDa; Monosaccharides (molar ratio): fructose:glucose = 28:1; - **Structures:** inulin-type fructan (β-2,1)	- **Physical barrier:** TJPs (ZO-1, Occludin), MUC2; sIgA	- **Barrier repair:** ↑ ZO-1/Occludin/MUC2/sIgA, ↓ permeability; -**Immune regulation:** microbiota-independent immunomodulation (↓ IL-6/IL-18/IL-17A/IL-1β/TNF-α/IFN-γ; ↑ IL-10; improved Th17/Treg); - **Inflammation pathways:** ↓ MAPK and ↓ NF-κB p65 nuclear translocation	(77)
Safflower polysaccharides (SPS)	Safflower	- *In vivo*: DSS-induced acute UC mouse model	- **Monosaccharides:** Arabinose, glucose, galactose, inositol acetate	- **Physical barrier:** TJPs (ZO-1, Occludin, Claudin-1), MUC1, MUC2, goblet cells	- **Barrier repair:** ↑ TJPs (ZO-1, Occludin, Claudin-1) & ↑ MUC1/MUC2 & ↑ goblet cells; Inflammation pathways: ↓ STAT3/NF-κB; -**Microbiota shift:** ↑ Akkermansia/Limosilactobacillus/Bifidobacterium; ↓ Bacteroides; - **Cytokine balance:** ↓ IL-6/TNF-α/IL-1β/L-8/IL-17/IL-20/IFN-γ/CHI3L1; ↑ IL-10/IL-22	(80)
Astragalus polysaccharides (APS)	Astragalus	- *In vitro*: LPS-RAW264.7; Caco-2; - *In vivo*: DSS-induced colitis mouse model	Not specified	- **Physical barrier:** TJPs (Occludin, Claudin-1); epithelial migration	- **Barrier repair:** ↑ TJPs (Occludin, Claudin-1); ↑ Caco-2 migration; -**Metabolic pathways:** ↑ SIRT1/PGC-1α/FXR (mitochondrial function); ↓ oxidative stress; - **Inflammation:** ↓ TLR4/MyD88/NF-κB (↓ p-p65); ↓ NO/TNF-α/IL-6/IL-1β	(9)
*Poria cocos* polysaccharides (PCP)	*P. cocos*	- *In vitro*: FH535 validation - *In vivo*: SPF C57BL/6 mice	- **Mw** 11.583 kDa (Mw/Mn=1.19); **Monosaccharides (molar ratio):** Mannose:D-glucosamine hydrochloride:glucose:galactose:fucose = 15.308:0.967:28.723:31.631:23.371; - **Structures:** the main chain contains t-Gal (p), 6-Gal (p), and 2,6-Gal (p).	- **Physical barrier:** TJPs (ZO-1, Occludin), MUC2, β-defensin	- **Pathway activation:** ↑ Wnt/β-catenin (↑ Wnt/Lrp5/β-catenin; blocked by FH535); ↑ ZO-1/Occludin/MUC2/β-defensin; -**Microbiota shift:** ↑ Muribaculaceae and ↑*Bacteroides*, ↓ Firmicutes,↑α-diversity; -**Metabolic synergy:** ↑ acetate/propionate; -**Immune rebalance:** ↑ IL-2/IL-4/IL-6/IL-10/TGF-β/IFN-γ	(37)
Sea buckthorn polysaccharides (SBP)	Sea buckthorn	- *In vivo*: DSS-induced acute colitis mouse model	- **Mw** 58.78 kDa; - **Monosaccharides (molar ratio):** Fructose: Rhamnose: Arabinose: Galactose: Glucose: Xylose: Galacturonic acid = 0.65: 5.97: 53.43: 6.05: 6.50: 2.59: 24.82	- **Physical barrier:** TJPs (ZO-1, Claudin-1), MUC2; - **Immune barrier:** Mucosal immune-related cytokines	- **Barrier repair:** ↑ ZO-1/Claudin-1/MUC2; -**Immune rebalance:** Th17/Treg rebalance (↓ IL-17F/RORγt, ↑ IL-10/TGF-β/Foxp3); -**Inflammation pathways:** ↓ IκB/NF-κB (↓ p-IκB/↓ p-NF-κB); -**Microbiota shift:** ↑*Ligilactobacillus*/*Lactobacillus*/*Bacteroides/Akkermansia, ↓ Escherichia–Shigella*/Lachnospiraceae_NK4A136_group/uncultured_Lactobacillus_sp.; -**Metabolic synergy:** ↑ SCFAs; bile acids ↑ CA/DCA/LCA	(102)
Rattan pepper polysaccharides (RPP)	Rattan pepper	- *In vivo*: DSS-induced colitis mouse model	- **Monosaccharides (molar ratio):** Arabinose:galactose:glucose:galacturonic acid:rhamnose = 30.46:34.50:15.49:10.40:5.07	- **Physical barrier:** TJPs (ZO-1, Claudin-1), MUC2	- **Barrier repair:** ↑ ZO-1/Claudin-1/MUC2; -**Microbiota shift:** ↑ Coriobacteriaceae_UCG-002, ↑ Lachnospiraceae_NK4A136_group, ↑*Desulfovibrio*; ↓*Parasutterella*, ↓*Olsenella*; -**Metabolic synergy:** ↑ SCFAs; bile acids ↑ DCA/LCA/UDCA; -**Gut-brain axis regulation:** ↓ TLR4/NF-κB; ↑ CREB/BDNF axis	(103)
Pumpkin polysaccharides (PPs)	Pumpkin	- *In vitro*: LPS/Nigericin-THP-1; - *In vivo*: DSS-induced UC mouse model	- **Mw** 31 kDa; **Monosaccharides (molar ratio):** Mannose:Rhamnose:Galacturonic acid:Galactosamine:Glucose:Xylose = 1.58:3.51:34.54:1.00:3.25:3.02; - **Structures:** homogeneous polysaccharide containing α-1,4-galacturonic acid as the main chain	- **Physical barrier:** TJPs (ZO-1, Claudin-1), MUC2; - **Immune barrier:** intestinal mucosal immune cytokine network	- **Barrier repair:** ↑ ZO-1/Claudin-1/MUC2, ↓ permeability; -**Microbiota shift:** ↑ Lachnospiraceae_NK4A136_group/*Helicobacter*, ↓ Bacteroidetes/Deferribacteres/Proteobacteria/*Escherichia–Shigella*; restored Firmicutes/Bacteroidetes;- **Metabolic synergy:** ↑ endogenous 5-HIAA (LC–MS/MS); - **Inflammation/Immune regulation:** ↑ PPARγ nuclear translocation; ↓ MAPK (↓ p38/ERK/JNK) and ↓ NF-κB; ↓ TNF-α/IL-1β/IL-6; ↑ IL-4/IL-10	(16)

Akt/AKT, protein kinase B; APS, *Astragalus membranaceus* polysaccharides; CHI3L1, chitinase-3-like protein 1; DSS, dextran sulfate sodium; E-cadherin, epithelial cadherin; FKBP5, FK506 binding protein 5; FXR, farnesoid X receptor; GPR43, G protein-coupled receptor 43; IEC-6, rat intestinal epithelial cell line IEC-6; IL, interleukin; JAK2, Janus kinase 2; LPS, lipopolysaccharide; MAPK, mitogen-activated protein kinase; MUC2, mucin 2; MyD88, myeloid differentiation primary response protein 88; NF-κB, nuclear factor kappa-B; PGC-1α, peroxisome proliferator-activated receptor-γ coactivator-1α; PCP, *Poria cocos* polysaccharides; PCYP, *Polygonatum cyrtonema* polysaccharides; qRT-PCR, quantitative reverse transcription PCR; RAW264.7, mouse macrophage-like cell line RAW264.7; SCFAs, short-chain fatty acids; SIRT1, sirtuin 1; sIgA, secretory immunoglobulin A; SPS, safflower polysaccharides; STAT3, signal transducer and activator of transcription 3; TJPs, tight junction proteins; TLR4, Toll-like receptor 4; UC, ulcerative colitis; ZO-1, zonula occludens-1.

#### 4.2.1 Restoration of the mechanical Barrier

Repair of the mechanical barrier centers on structural remodeling. The intestinal mechanical barrier consists of a single layer of columnar epithelial cells that fulfills two key functions: first, precise regulation of the transport of luminal contents such as nutrients, water, and electrolytes into the circulation; second, prevention of abnormal translocation of luminal toxins, microorganisms including commensals and pathogens, and antigens (69). The homeostasis of this barrier relies heavily on the tight junction complex, comprising transmembrane proteins (such as claudins, occludin, and tricellulin) and cytoplasmic adaptor proteins (such as the ZO family), whose expression levels and membrane localization directly determine the sealing capacity of the epithelium (70, 71).

A number of dietary polysaccharides have been shown to enhance barrier function by promoting tight junction expression and assembly. Tremella aurantialba polysaccharides, for example, significantly improve intestinal barrier integrity by upregulating claudin-1 and ZO-1 (72). Longan pulp polysaccharides not only increase tight junction proteins such as ZO-1, claudin-1, and claudin-4, but also upregulate the expression of the adherens junction protein E-cadherin, thereby strengthening intercellular contacts on multiple levels and preventing mucosal injury (17).

Under pathological conditions, pro-inflammatory cytokines such as TNF-α induce selective barrier disruption, including overexpression of pore-forming claudin-2 and internalization and ubiquitin-mediated degradation of ZO-1(73). To counteract these damaging mechanisms, the low-molecular-weight fraction of Astragalus polysaccharides exhibits multi-targeted repair activity: it downregulates claudin-2 expression by inhibiting the NF-κB signaling pathway and reduces the ubiquitination-mediated degradation of ZO-1, thereby stabilizing the junctional complex (9).

The actin cytoskeleton is another core determinant of barrier integrity. Cortical F-actin regulates cortical tension and is essential for maintaining cell shape, adherens junctions, and the proper localization of junctional proteins, while the Ras Homolog Family Member A-Rho-associated protein kinase (RhoA-ROCK1) signaling pathway is a major upstream regulator of this process (74). Zhenxing Wang and colleagues reported that yam polysaccharides can restore the structural and functional integrity of damaged intestinal barriers by inhibiting the RhoA-ROCK pathway activity, promoting F-actin polymerization, and increasing the expression of tight junction proteins (12).

#### 4.2.2 Reinforcement of the chemical barrier

Reinforcement of the chemical barrier involves functional upgrading of the active defense system. In the colon, the mucus layer forms the first protective interface covering the gastrointestinal surface. It physically separates the epithelium from the intestinal lumen and shields epithelial cells from direct exposure to bacteria, microbial metabolites, and dietary antigens (75). The mucus layer is composed of approximately 30 core proteins, including mucins, antimicrobial peptides, and secretory immunoglobulin A. Goblet cells are central effector cells of the chemical barrier, and the gel forming mucin MUC2 synthesized and secreted by these cells is the main structural component of colonic mucus (76).

This mucus layer acts as a physical barrier that spatially segregates bacteria from the epithelium. At the same time, the abundant glycosylation sites on mucin molecules can competitively bind to pathogenic bacteria, interfering with their adhesion to the gut surface and thereby serving as a chemical barrier against colonization (77). Several studies have shown that dietary polysaccharides are effective modulators of mucus barrier function. GLP significantly promote goblet cell proliferation and differentiation and increase MUC2 expression and secretion, resulting in thickening of the mucus layer (65). *Polygonatum cyrtonema* polysaccharides have similarly been shown to restore MUC2 expression in experimental colitis models and to repair inflammation induced damage to the chemical barrier (78).

Antimicrobial peptides secreted by intestinal epithelial cells, particularly Paneth cells, represent another key component of the chemical barrier. These peptides, including defensins, lysozyme, and the regenerating islet derived protein family regenerating islet-derived protein 3 (Reg3), are critical effector molecules of innate immunity. They exert rapid antimicrobial activity through diverse mechanisms such as disrupting bacterial membranes and inhibiting vital metabolic processes (79, 80). Safflower polysaccharides enhance this defense system by stimulating the secretion of MUC1 and MUC2 and inducing the expression of Reg3 family antimicrobial peptides, thereby strengthening the chemical barrier against microbial invasion (81).

#### 4.2.3 Reduction of intestinal permeability

The ultimate outcome of structural and functional repair of physical and chemical barriers is reduced epithelial permeability. This process is closely linked to coordinated regulation of apoptosis and endoplasmic reticulum stress. The intestinal epithelium is a highly dynamic tissue in which proliferation, differentiation, and cell death must be tightly regulated. When this balance is disturbed or apoptosis is excessive, severe intestinal disease can result. For example, colonic biopsy samples from patients with CD exhibit approximately twofold higher levels of epithelial apoptosis than those from healthy controls (82).

Several polysaccharides have demonstrated protective effects by suppressing apoptosis. APS-G2 helps maintain barrier integrity in DSS induced colitis by preventing epithelial apoptosis and enhancing tight junction protein expression (9). Soy polysaccharides significantly inhibit activation of the pro apoptotic protein caspase-3 in mice, thereby reducing programmed cell death of intestinal epithelial cells and preserving epithelial continuity (68).

Alleviation of endoplasmic reticulum stress is another important mechanism underlying barrier repair. Inhibition of the inositol-requiring enzyme 1α-X-box binding protein 1 pathway can reduce overactivation of the unfolded protein response, restore intracellular proteostasis, and prevent barrier dysfunction driven by cellular stress (83). LBP have been shown to markedly attenuate endoplasmic reticulum stress by modulating this pathway (84). Together, these structural repair and functional protection strategies facilitate coordinated reconstruction of both physical and chemical barriers, ultimately lowering epithelial permeability and promoting comprehensive restoration of intestinal homeostasis.

#### 4.2.4 Promotion of intestinal epithelial regeneration

Beyond the repair of existing cellular junctions and the mucus layer, the regeneration of intestinal epithelial cells is also crucial for the treatment of IBD. The intestinal epithelium undergoes rapid renewal every 3–5 days to maintain the epithelial barrier and homeostasis, a process driven by intestinal stem cells (ISCs) located at the base of the crypts, with Lgr5 identified as a marker of ISCs (85). In IBD, chronic inflammation induces ISC apoptosis and disrupts crypt architecture, thereby impairing epithelial renewal (86). Emerging evidence suggests that dietary polysaccharides can promote epithelial regeneration by targeting ISCs and their niche (68, 87, 88).

Numerous polysaccharides have been demonstrated to promote ISCs-mediated regeneration through the IL-22 pathway, albeit via distinct mechanisms (87, 89). For instance, a polysaccharide derived from Dendrobium fimbriatum (cDFPW1) has been shown to significantly increase the number of Lgr5+ cells in both intestinal organoids and the colonic mucosa of mice. Mechanistic studies have revealed that cDFPW1 stimulates lamina propria lymphocytes (LPLs) to secrete IL-22, which in turn promotes ISC proliferation and preserves mucosal integrity (87). Similarly, Astragalus polysaccharides (APS) facilitate ISC regeneration by activating the IL-22 axis, but through a different upstream mechanism: APS enriches the abundance of Lactobacillus in the gut microbiota, which subsequently induces IL-22 signaling activation (89). Beyond its effects on IL-22 signaling, APS also activates the hypoxia-inducible factor 1 (HIF-1) pathway—a signaling cascade critical for ISC function and mucosal homeostasis. Notably, pharmacological inhibition or genetic ablation of HIF-1 completely abolishes the radioprotective and pro-regenerative effects of APS, indicating that HIF-1 is a key mediator in APS-induced ISC repair (90).

Gut microbiota-derived metabolites also contribute to polysaccharide-induced regeneration. Studies have shown that soy polysaccharide (SP) enriches Lactobacillus and increases luminal lactate production. Lactate activates the Gpr81 receptor on intestinal epithelial cells, triggering Wnt3/β-catenin signaling and promoting ISCs expansion (68). In addition to classical microbial metabolites such as short-chain fatty acids and lactate, bile acids have emerged as another important class of intestinal metabolites involved in the regulation of ISC activity. Sorrentino et al. demonstrated that bile acids activate the G protein-coupled receptor TGR5 on ISCs, initiating downstream SRC-YAP1 signaling, which significantly enhances ISC proliferative activity and self-renewal capacity, thereby accelerating intestinal epithelial homeostasis and post-injury regeneration (91).

### 4.3 Modulation of immune inflammation: rebuilding immune homeostasis

Excessive activation of the intestinal immune system and loss of immune homeostasis are major driving forces behind the onset and persistence of IBD. Rebuilding immune homeostasis requires multidimensional reprogramming of immune networks to achieve a precise shift from hyperinflammatory states toward balanced responses (92). Dietary polysaccharides such as *Astragalus* polysaccharides (93), Crataegus pinnatifida polysaccharides (94), and GLP (95) exhibit broad immunomodulatory properties. They not only enhance host immunity but also suppress exaggerated immune responses induced by various stimuli by targeting multiple nodes of cytokine signaling, immune cell function, and intracellular pathways (96). Table 3 summarizes the compositional characteristics and pharmacological information of dietary-derived polysaccharides used for the treatment of IBD through immunomodulatory mechanisms.

**Table 3:** Representative dietary polysaccharides that modulate immune inflammation and rebuild immune homeostasis in experimental colitis/IBD models.

Polysaccharides	Dietary source	Experimental models	Chemical/molecular features	Mechanism	References
Quinoa polysaccharides (QPS)	Quinoa	- *In vitro*: cell-free *in vitro* model - *In vivo*: LPS-induced inflammatory mouse model	- **Mw:** Q1, 4.283 × 104 Da; Q2, 4.88 × 103 Da; - **Monosaccharides:** Q1 contained xylose, arabinose, rhamnose, glucose, galactose, and galacturonic acid, with a molar ratio of 16.968:44.333:9.817:11.686:5.166:12.026; Q2 contained glucose and galacturonic acid, with a molar ratio of 82.693:17.306; - **Polysaccharide content:** 91.75%	- ↓IL-6, TNF-α, IL-1β; ↑IL-10; - ↑SOD, T-AOC; ↓MDA; - Improved immune organ indices and enhanced immune function	(94)
Hawthorn polysaccharides (HAW1–2)	Hawthorn (*Crataegus pinnatifida*)	- *In vitro*: LPS-induced IEC-6 cell model; - *In vivo*: DSS-induced colitis mouse model	- **Mw:** 8.94 kDa; **Monosaccharides:** arabinose, galactose, glucose; - **Structures:** heteropolysaccharide composed of arabinose, galactose, and glucose	- ↓phosphorylation of IKKα/β, IκBα, NF-κB; - ↓IL-1β, IL-6, TNF-α	(86)
Alkali-soluble purple sweet potato polysaccharide (ASPP)	Purple sweet potato	- *In vivo*: DSS-induced colitis mouse model	- **Mw:** 180 kDa; - **Monosaccharides:** glucose, mannose, rhamnose, arabinose, xylose (53.3:7.6:2.8:1.9:1.0); - **Structure:** 1,4-α-D-glucan backbone with extensive branching at O-6	- ↓IL-6, TNF-α, IL-1β; - improved immune organ indices and immune regulation	(95)
*Cistanche deserticola* polysaccharides (CDPS)	*Cistanche deserticola*	- *In vivo*: DSS-induced IBD mouse model	- **Mw:** 3.31–1019.07 kDa; - **Monosaccharides:** glucose (63.27%), arabinose (15.56%), galactose (9.10%)	- ↓IL-6, IL-1β, TNF-α; ↑IL-10; - ↓activation of SRC/EGFR/PI3K/AKT pathway; - ↓MPO activity and inflammatory cell infiltration	(97)
*Poria cocos* polysaccharides (PCP)	*Poria cocos*	- *In vitro*: FH535 validation - *In vivo*: SPF C57BL/6 mice	- **Mw** 11.583 kDa (Mw/Mn=1.19); **Monosaccharides (molar ratio):** Mannose:D-glucosamine hydrochloride:glucose:galactose:fucose = 15.308:0.967:28.723:31.631:23.371; - **Structures:** the main chain contains t-Gal (p), 6-Gal (p), and 2,6-Gal (p).	- ↑IL-2, IL-4, IL-6, IL-10, TGF-β, IFN-γ; - ↑sIgA; - ↑ expression of MUC2 and β-defensins; - regulated neurotransmitter-related immune balance	(37)
Astragalus polysaccharides (APS)	*Astragalus*	- *In vitro*: LPS-RAW264.7; Caco-2; - *In vivo*: DSS-induced colitis mouse model	Not specified	- ↓TNF-α, IL-6, IL-1β, IL-21, p-p65 and NO release; ↑IL-10, TGF-β; - ↑Treg proportion/function (↑Foxp3, IL-10); ↓Bcl-6/Blimp-1; - ↓TLR4/MyD88/NF-κB; ↑SIRT1/PGC-1α/FXR axis (improved mitochondrial function, reduced oxidative stress); - ↓Tfh cells & pro-inflammatory subsets (Tfh1/17/21); ↑anti-inflammatory subsets (Tfh10/Tfr)	(9, 14, 85)
Sea buckthorn polysaccharides (SBP)	Sea buckthorn	- *In vivo*: DSS-induced acute colitis mouse model	- **Mw** 58.78 kDa; - **Monosaccharides (molar ratio):** Fructose: Rhamnose: Arabinose: Galactose: Glucose: Xylose: Galacturonic acid = 0.65: 5.97: 53.43: 6.05: 6.50: 2.59: 24.82	- ↓IL-6, IL-1β, TNF-α, IL-17F; ↑IL-10, TGF-β; - regulated Th17/Treg balance (↓RORγt, ↑Foxp3); - ↓IκB/NF-κB activation (↓p-IκB, p-NF-κB)	(102)
Rattan pepper polysaccharides (RPP)	Rattan pepper	- *In vivo*: DSS-induced colitis mouse model	- **Monosaccharides (molar ratio):** Arabinose:galactose:glucose:galacturonic acid:rhamnose = 30.46:34.50:15.49:10.40:5.07	- ↓TNF-α, IL-1β, IL-6; ↓COX-2, iNOS; - regulated Th17/Treg (↑Foxp3, ↓RORγt); - ↓TLR4/NF-κB; activated CREB/BDNF pathway	(103)
*Ganoderma lucidum* polysaccharides (GLP)	*Ganoderma lucidum*	- *In vitro*: BMDM model; RAW264.7 macrophage model; - *In vivo*: DSS-induced UC mouse model	- **Mw** 25.0 kDa; -**Monosaccharides:** β-glycosidic bonds; glucose 78.15%, mannose 15.69%; - **Structures:** Water-soluble heteropolysaccharide containing β-glycosidic bonds	- ↓IL-17, IL-1β, IL-6 and Th17 TF RORγT; ↑FOXP3, IL-10; - macrophage polarization (↑M2 markers Arg-1, Ym1; ↓M1 markers CD80, NOS2); - Notch-pathway–mediated macrophage regulation; - ↑phagocytosis and innate immune response	(87, 105)
*Lycium barbarum* polysaccharides (LBP)	Goji berry (*Lycium barbarum*)	- *In vitro*: Probiotic culture model (Lactobacillus acidophilus, Bifidobacterium longum); RAW264.7 macrophage polarization model (LPS/IFN-γ-induced M1; IL-4/IL-13–induced M2); - *In vivo*: Kunming mouse model (oral gavage for 14 days); DSS-induced IBD mouse model	- **Monosaccharides:** glucose (molar proportion 6.52); arabinose:rhamnose:xylose:mannose:galactose = 0.18:0.81:0.07:2.17:0.23 (molar ratio), total ≈ 10	- ↓IL-6, TNF-α, IL-1β; ↑IL-10, TGF-β, sIgA; - ↓M1 (↓NOS2); ↑M2 (↑Arg-1); - ↓STAT1 phosphorylation; ↑STAT6 phosphorylation	(11, 104)
Apple polysaccharides (AP)	Fuji apple	- *In vitro*: LPS-induced RAW264.7 macrophage inflammation model	- **Mw:** 7.9 kDa; -**Monosaccharides (molar ratio):** rhamnose, galactose, arabinose, glucose, galacturonic acid (4.3:5.2:2.6:1.0:11.9); - **Structures:** α-Galactose -(1 → [3)-α-Rhamnose -(1 → 2)-α-Rhamnose -(1]_2_ → [4)-α-Galacturonic acid -(1]10 → 3,6)-β-Glucose -(1 → 6)-β-Glucose -(1 → 4)-β-Galactose -(1 → 4)-β-Galactose -(1 → The main chain contains arabinose side chains)	- ↓TNF-α, IL-1β, IL-6; ↑IL-10; - ↓ROS, NO and iNOS/COX-2; - targeted TLR4/NF-κB (↓p-NF-κB, p-IκBα; ↓IκBα degradation)	(36)
Almond polysaccharides (AP-1)	Almond	- *In vitro*: LPS-induced RAW264.7 inflammation model - *In vivo*: DSS-induced UC mouse model	- **Monosaccharides:** glucose, arabinose, galactose, mannose; - **Structures:** triple-helix; heteropolysaccharide	- ↓TNF-α, IL-1β, IL-6; ↑IL-10; - ↓NF-κB activation (↓p-p65, p-IκBα); - ↓MPO, NO; ↑T-SOD, GSH-Px	(106)
Pumpkin polysaccharides (PPs)	Pumpkin	- *In vitro*: LPS/Nigericin-induced THP-1 inflammation model; - *In vivo*: DSS-induced UC mouse model	- **Mw** 31 kDa; -**Monosaccharides (molar ratio):** Mannose:Rhamnose:Galacturonic acid:Galactosamine:Glucose:Xylose = 1.58:3.51:34.54:1.00:3.25:3.02; - **Structures:** homogeneous polysaccharide containing α-1,4-galacturonic acid as the main chain	- ↓TNF-α, IFN-γ, IL-1β; ↑IL-4, IL-10; - activated PPARγ nuclear translocation; ↓MAPK/NF-κB; - ↑endogenous 5-HIAA	(16)
*Glycyrrhiza* polysaccharides (GP)	Licorice (*Glycyrrhiza*)	- *In vivo*: CTX-induced immunosuppression + intestinal mucosal injury mouse model; FMT validation model	- **Mw** 6.5 kDa; - **Structures:** contains 1,6-linked glucose residues; low-molecular-weight heteropolysaccharide	- maintained Th1/Th2 balance (↑T-bet, GATA-3 mRNA); - ↓ERK/p38/NF-κB p50; ↑IL-2/IFN-γ/IL-4; - ↑CD4^+^/CD8^+^ T cells and IgM/IgG/sIgA secretion	(107)
*Asparagus cochinchinensis* polysaccharides (ACMP)	*Asparagus cochinchinensis*	- *In vitro*: LPS-induced RAW264.7 inflammation model; BMDM trained-immunity model	- **Monosaccharides:** xylose, galactose, galacturonic acid, mannose, glucose, etc. (97.12:3.58:1.00:1.41:0.36, etc.); - **Structure:** heteropolysaccharide with pyranose residues	- induced H3K4me1 histone modification; ↑TNF-α, IL-1β; - regulated immunity via TLR4–MAPK (JNK/p38/ERK); - promoted innate immune memory and enhanced secondary responses	(108)
*Dendrobium officinale* polysaccharides (DOPS)	*Dendrobium officinale*	- *In vitro*: LPS-stimulated NCM460 inflammation model; - *In vivo*: DSS-induced acute UC mouse model;	- **Mw:** 393.8 kDa; - **Monosaccharides:** mannose, glucose, arabinose (5.55:1:0.12); 2) Mw: 393.8 kDa; - **Structure:** mainly 2-O-acetyl glucomannan backbone	- ↓IL-1β, IL-6, TNF-α; ↑IL-10; - ↓NLRP3 inflammasome activation (↓ASC, caspase-1); - ↓β-arrestin1 protein	(109)
*Hericium erinaceus* polysaccharides (HECP)	*Hericium erinaceus*	- *In vivo*: DSS-induced UC mouse model	- **Mw:** 86.67 kDa; - **Structure:** homogeneous polysaccharide containing glucan components	- ↓TNF-α, IL-1β, IL-6; ↓iNOS, COX-2; - blocked phosphorylation of NF-κB, PI3K/Akt, MAPK (p38/ERK/JNK); - ↓NO, MDA; ↑T-SOD	(49)
*Gastrodia elata* polysaccharides (GEP/GBP)	*Gastrodia elata*	- *In vitro*: Simulated saliva–gastric–intestinal digestion + *in vitro* fermentation with human feces; - *In vivo*: DSS-induced IBD mouse model	- **Mw:** digestion products (GEP-I) 41.328 kDa and 1.801 kDa; purified fractions GBP1 (1.435 × 106 g/mol), GBP2 (1.913 × 106 g/mol); - **Monosaccharides:** mainly glucose (90.56%−98.95%), with galactose, GalA, rhamnose, arabinose, GlcA, etc.; - **Structure:** backbone includes α-1,4-glucan, α-1,4,6-glucan, β-1,6-glucan, etc.	- ↓IL-1β, TNF-α, IL-6; ↑tryptophan metabolism and vitamin B6 metabolism; - regulated Th17/Treg balance and improved immune microenvironment	(52, 53)
*Codonopsis pilosula* polysaccharides (CPPS)	*Codonopsis pilosula*	- *In vivo*: DSS-induced UC mouse model; FMT model	- **Mn** 6.056 kDa; **Mw** 29.386 kDa; **PDI** 4.852; - **Monosaccharides:** rhamnose 3.82%, arabinose 24.67%, galactose 10.11%, glucose 10.31%, galacturonic acid 51.10%	↓IL-1β, IL-18, IL-6, TNF-α; ↓NLRP3 inflammasome activation	(15)
Oat β-glucan (BG)	Oat	- *In vivo*: DSS-induced UC mouse model	- **Mw** 614.543 kDa (homogeneous); -**Monosaccharides:** 97.47 mol% glucose; - **Structures:** linear β-glucan with β-(1 → 3) and β-(1 → 4) linkages; purification not specified	↓TNF-α, IFN-γ, IL-6	(48)
Turmeric polysaccharides (TPS)	Turmeric	- *In vivo*: DSS-induced UC mouse model	- **Monosaccharides (molar ratio):** ribose:xylose:fructose:glucose:galactose = 5.8:0.8:19.3:49.4:24.7	- Activated AhR; ↑IL-22; (as written) ↓TNF-α, IL-17, IL-10; - ↓CD45^+^ immune cell and F4/80^+^ macrophage infiltration; - ↑hepatic GSH/GSSG ratio	(58)
Polygonatum polysaccharides (PCYP)	Polygonatum (Huangjing)	- *In vitro*: Caco-2/RAW264.7 co-culture - *In vivo*: DSS-induced UC mouse model; antibiotic-depleted model	- **Mw** 5.65 kDa; Monosaccharides (molar ratio): fructose:glucose = 28:1; - **Structures:** inulin-type fructan (β-2,1)	- ↓IL-6, TNF-α, IL-18, IL-17A, IFN-γ, IL-1β; ↑IL-10; gut microbiota–independent effect persists after antibiotics; - regulated Th17/Treg (↓Th17 and IL-17/RORγt; ↑Treg and FOXP3/IL-10); - ↓MAPK (ERK/JNK/p38) phosphorylation and NF-κB p65 nuclear translocation	(77)
Safflower polysaccharides (SPS)	Safflower	- *In vivo*: DSS-induced acute UC mouse model	- **Monosaccharides:** Arabinose, glucose, galactose, inositol acetate	- ↓IL-6, TNF-α, IL-1β, IL-8, IL-17, IL-20 and IFN-γ; ↑IL-10, IL-22; - ↓STAT3/NF-κB (↓p-STAT3, p-NF-κB p65); - ↓CHI3L1 mRNA and protein	(80)

AhR, aryl hydrocarbon receptor; Akt/AKT, protein kinase B; AP, apple polysaccharides; AP-1, activator protein-1; Arg-1, arginase-1; ASPP, *Asparagus officinalis* polysaccharides; BDNF, brain-derived neurotrophic factor; BMDM, bone marrow-derived macrophages; COX-2, cyclooxygenase-2; CREB, cAMP response element-binding protein; DSS, dextran sulfate sodium; Foxp3, forkhead box P3; GLP, *Ganoderma lucidum* polysaccharides; GPX4, glutathione peroxidase 4; GSH, reduced glutathione; GSH-Px, glutathione peroxidase; GSSG, oxidized glutathione; H3K4me1, histone H3 lysine 4 monomethylation; HAW1–2, *Crataegus pinnatifida* polysaccharide HAW1–2; HECP, *Hericium erinaceus* polysaccharides; IκBα, inhibitor of NF-κB alpha; IFN-γ, interferon-gamma; IL, interleukin; iNOS, inducible nitric oxide synthase; IKKα/β, IκB kinase α/β; LBP, *Lycium barbarum* polysaccharides; LPS, lipopolysaccharide; M1/M2, classically/alternatively activated macrophages; MDA, malondialdehyde; MPO, myeloperoxidase; NF-κB, nuclear factor kappa-B; NOS2, nitric oxide synthase 2; PCP, *Poria cocos* polysaccharides; PI3K, phosphoinositide 3-kinase; PPs, pumpkin polysaccharides; Reg3, regenerating islet-derived protein 3; RORγt, RAR-related orphan receptor gamma t; ROS, reactive oxygen species; RPP, rattan pepper polysaccharides; SCFAs, short-chain fatty acids; STAT1, signal transducer and activator of transcription 1; STAT6, signal transducer and activator of transcription 6; T-AOC, total antioxidant capacity; T-SOD, total superoxide dismutase; Tfh, T follicular helper cell; TGF-β, transforming growth factor-β; Th17, T helper 17; TNF-α, tumor necrosis factor-α; Treg, regulatory T cell; Ym1, chitinase-like protein 3 (Chil3).

#### 4.3.1 Remodeling of cytokine networks

Cytokines are small signaling proteins secreted by immune cells including lymphocytes, macrophages, and natural killer cells. They regulate proliferation, differentiation, apoptosis, and immune responses via receptor-mediated signaling cascades and together form a dynamic and interconnected cytokine network (97). A hallmark of IBD is disruption of this network, characterized by overproduction of pro-inflammatory cytokines (98).

TNF-α is a central cytokine in IBD that promotes infiltration of macrophages and neutrophils into the intestinal mucosa, increases mucosal permeability, and reduces tight junction protein expression, thereby compromising barrier integrity (99). Members of the IL family also play key roles in immune dysregulation. IL-6 is mainly produced by macrophages and CD4-positive T cells and promotes polarization of macrophages toward the pro-inflammatory M1 phenotype, enhances cytokine release, and interferes with forkhead box P3 (Foxp3) interaction with the chromatin modifying enzyme enhancer of zeste homolog 2, thereby impairing regulatory T cell function and exacerbating inflammation (100). IL-1β, the major effector cytokine of the NLRP3 inflammasome, directly contributes to the initiation and amplification of inflammatory responses (101).

Many dietary polysaccharides can effectively suppress the aberrant expression of these pro-inflammatory mediators. Quinoa polysaccharides reduce IL-6 and IL-1β production in LPS stimulated macrophages and *in vivo* (102). Crataegus pinnatifida polysaccharides HAW1-2 (94) and an alkali-soluble polysaccharide from purple sweet potato (103) significantly lower the levels of IL-1β, IL-6, and TNF-α in colonic tissue and serum, attenuate histopathological damage, and alleviate inflammation.

In parallel with inhibition of pro-inflammatory cytokines, dietary polysaccharides also promote the expression of anti-inflammatory and reparative cytokines, thereby creating a microenvironment conducive to resolution of inflammation and tissue regeneration. IL-10 is a key anti-inflammatory cytokine that limits inflammation by activating STAT3 signaling, suppressing reactive oxygen species production, and downregulating pro-inflammatory mediators such as IL-8 and monocyte chemoattractant protein-1 (104). *Cistanche deserticola* polysaccharides have been reported to elevate IL-10 expression in colonic tissue, thus enhancing anti-inflammatory capacity (105).

Transforming growth factor β (TGF-β) is another multifunctional cytokine, particularly TGF-β1, which, upon binding to its receptor, activates Smad2/3 signaling. This pathway promotes epithelial repair and extracellular matrix remodeling and directly suppresses excessive activation of immune cells, thereby playing a central role in inflammation resolution and tissue healing (106). *Poria cocos* polysaccharides broadly modulate cytokine networks and increase the expression of IL-2, IL-4, IL-10, and TGF-β(37). *Astragalus* polysaccharides demonstrate remarkable bidirectional regulatory effects in DSS induced colitis by significantly suppressing pro-inflammatory cytokines such as IL-6 and TNF-α while upregulating TGF-β1 and IL-10 levels in the colonic mucosa (93). This dual action allows more effective restoration of cytokine balance than single-target biologic agents.

#### 4.3.2 Rebalancing of immune cell subsets

Beyond cytokine modulation, a second key mechanism by which dietary polysaccharides exert anti-inflammatory effects involves functional rebalancing of critical immune cell populations. This process mainly targets T lymphocytes of the adaptive immune system and macrophages of the innate immune system.

The balance between Th17 cells and Treg cells is essential for intestinal immune homeostasis. Th17 cells, driven by the transcription factor RAR-related orphan receptor gamma t (RORγt), produce pro-inflammatory cytokines such as IL-17A and IL-17F and are induced from naive CD4-positive T cells under the influence of gut microbiota. They contribute to the pathogenesis of many autoimmune diseases including IBD (107). In contrast, Treg cells, characterized by expression of the transcription factor Foxp3, are critical suppressor cells that limit immune responses by inhibiting effector T cells, including Th17 cells, and by secreting IL-10 and other anti-inflammatory mediators (108). In IBD, Th17 responses are exaggerated while Treg function is impaired, resulting in a shift toward a pro-inflammatory state (109).

Synbiotics containing sea buckthorn polysaccharides restore Th17/Treg balance in DSS induced colitis. Treatment significantly reduces the expression of Th17 associated cytokines IL-6, IL-1β, TNF-α, and IL-17F and the transcription factor RORγt, while increasing Treg associated cytokines IL-10 and TGF-β and Foxp3 expression (110). Rattan pepper polysaccharides similarly rebalance Th17 and Treg cells, thereby stabilizing immune responses and ameliorating intestinal inflammation (111). Fungal polysaccharides such as those derived from *Ganoderma lucidum* appear to exhibit particularly strong modulatory activity in this context, suggesting that polysaccharides from different sources may act through preferential pathways (95). *Astragalus* polysaccharides not only increase Treg levels in colitic mice but also reduce T follicular helper (Tfh) cells and the expression of their key transcription factors Blimp-1 and Bcl-6 and effector cytokine IL-21. Through simultaneous modulation of Tfh and Treg populations, they help re-establish immune homeostasis and alleviate UC (14).

Macrophages are central components of innate immunity, and the dynamic balance between pro-inflammatory M1 and anti-inflammatory or tissue-repair-oriented M2 macrophages is crucial for both disease progression and resolution in IBD. Dietary polysaccharides can promote M1 to M2 repolarization through distinct mechanisms. LBP inhibit signal transducer and activator of transcription 1 phosphorylation that drives M1 polarization and enhance signal transducer and activator of transcription 6 phosphorylation that supports M2 differentiation, thereby favoring an M2 phenotype (112). *Ganoderma atrum* polysaccharides PSG-1 regulate macrophage polarization through both Notch dependent and Notch independent pathways, coordinating M1/M2 balance and exhibiting strong anti-inflammatory and tissue repair capacity (113).

#### 4.3.3 Targeting of signaling pathways

Targeted regulation of intracellular signaling pathways is a core molecular mechanism by which dietary polysaccharides help restore intestinal immune homeostasis. Their primary actions involve suppression of key pro-inflammatory pathways, particularly NF-κB, MAPK, and the NLRP3 inflammasome.

At the NF-κB level, multiple polysaccharides have demonstrated notable efficacy. Apple polysaccharides suppress TLR4 expression, inhibit degradation and phosphorylation of IκBα, and prevent nuclear translocation of NF-κB, thereby downregulating the transcription of TNF-α, IL-1β, IL-6, and other pro-inflammatory cytokines (36). Almond polysaccharides AP-1 directly reduce phosphorylation of NF-κB and IκBα and decrease the expression of downstream effector proteins such as inducible nitric oxide synthase (iNOS) and cyclooxygenase-2 (COX-2), effectively blocking inflammatory signaling at its source (114).

With respect to MAPK signaling, different polysaccharides suppress pathway activation through various upstream mechanisms. Pumpkin polysaccharides activate peroxisome proliferator-activated receptor γ, which in turn inhibits both MAPK and NF-κB pathways (16). Glycyrrhiza polysaccharides directly reduce phosphorylation of extracellular signal-regulated kinase (ERK) and p38 (115). *Asparagus cochinchinensis* polysaccharides exert broader control by targeting TLR4 and simultaneously inhibiting c-Jun N-terminal kinase, ERK, and p38 activation, thereby providing comprehensive inhibition of MAPK signaling (116).

The NLRP3 inflammasome has emerged as an important additional target because its overactivation acts as a trigger for acute intestinal inflammation. *Dendrobium officinale* polysaccharides specifically inhibit activation of the NLRP3 inflammasome and its upstream regulator β-arrestin1, thereby blocking caspase 1-mediated maturation and release of IL-1β and offering a promising strategy to prevent acute inflammatory flares (117).

Beyond single pathway targeting, many dietary polysaccharides display powerful multi-pathway regulatory potential. *Hericium erinaceus* polysaccharides provide a representative example. In DSS induced colitis, these polysaccharides concurrently inhibit phosphorylation of NF-κB, MAPK, and Akt signaling axes, thereby suppressing pro-inflammatory cytokine production and reducing expression of inflammatory mediators such as iNOS and COX-2 at multiple levels. This coordinated action results in robust anti-inflammatory efficacy (50).

### 4.4 Structure-activity trends of dietary polysaccharides in IBD models

Although dietary polysaccharides can alleviate IBD through the multi-target mechanisms described above, their biological activities are highly structure-dependent. Therefore, systematically elucidating the structure–function relationships of polysaccharides from different sources is essential for gaining deeper insight into their mechanisms of action and for guiding the rational selection and development of bioactive polysaccharides. This section focuses on key structural factors, including molecular weight (Mw), monosaccharide composition, glycosidic linkage patterns, and chemical modifications, and summarizes how these features influence IBD-related bioactivities.

#### 4.4.1 Molecular weight

Molecular weight is one of the key determinants of the intestinal bioavailability and biological activity of polysaccharides. In many reported cases, polysaccharides with relatively low to medium molecular weights tend to exhibit more favorable anti-IBD effects than higher-molecular-weight counterparts, although this trend is influenced by polysaccharide source and structural context. This is primarily attributed to their better water solubility, enhanced ability to penetrate the intestinal mucus layer and reach epithelial cells, as well as their greater susceptibility to fermentation by the gut microbiota, leading to the production of functional metabolites such as SCFAs (45). This pattern is supported by multiple studies. Li et al. investigated Astragalus polysaccharides and found that the low molecular weight fraction (Mw ≈ 10 kDa) effectively suppressed the NF–κB signaling pathway in the colonic tissue of DSS–induced colitis mice, reduced the ubiquitin–mediated degradation of the tight junction protein ZO−1, and thereby stabilized the intestinal mechanical barrier. In contrast, the high molecular weight fraction (Mw > 200 kDa) exhibited poor water solubility, low intestinal availability, and no significant protective activity (118). Similarly, Dou et al. demonstrated that low molecular weight blackberry polysaccharides (Mw < 50 kDa) were more readily fermented by the mouse gut microbiota, significantly increasing luminal SCFA levels and exerting prebiotic and anti–inflammatory effects, whereas the high molecular weight fraction showed substantially lower fermentation efficiency and biological activity (119). Reducing the molecular weight of citrus pectin (CP) to 18.18 kDa and okra pectin (OP) to 119.12 kDa was also associated with enhanced therapeutic efficacy (120).

#### 4.4.2 Monosaccharide composition

The monosaccharide composition of dietary polysaccharides is an important determinant of their biological effects in IBD. Converging evidence suggests that polysaccharides rich in arabinose and galactose, particularly arabinogalactans, are frequently associated with pronounced protective effects in IBD models (121). Wu et al. provided direct evidence for this functional specificity: lemon pectin, characterized by arabinan-rich side chains, primarily exerts anti-inflammatory effects by inhibiting the pro-inflammatory cytokine IL-6, whereas potato pectin, dominated by galactan side chains, preferentially modulates intestinal barrier function and enhances anti-inflammatory cytokine levels (122). This functional specificity has been corroborated by studies on arabinogalactans from other sources. An arabinogalactan isolated from banana was shown to significantly suppress the secretion of TNF-α and IL-1β while promoting IL-10 production. Mechanistically, these effects involve the regulation of NF-κB/MAPK/PPARγ signaling pathways and the inhibition of NLRP3 inflammasome activation; additionally, this polysaccharide modulates gut microbiota composition and butyrate metabolism (121, 123). Similarly, the homogeneous arabinogalactan LBP–m purified from goji berry exerts protective effects through two complementary mechanisms: on one hand, it activates the Nrf2/HO-1 pathway to enhance antioxidant capacity and inhibits the NF-κB pathway to alleviate mucosal inflammation; on the other hand, it reshapes gut microbiota composition, promotes short-chain fatty acid production, and upregulates tight junction protein expression to restore intestinal barrier function (124).

#### 4.4.3 Glycosidic linkage types and main-chain structural domains

The type of glycosidic linkage determines the main-chain conformation and spatial structure of polysaccharides, while the composition of the core structural domains directly influences their degradation patterns and recognition by immune cells. Together, these two features form an important basis for the structure–activity relationships of polysaccharides. β-type glycosidic linkages are an important structural feature underlying the IBD-protective activity of polysaccharides. On one hand, these linkages are resistant to hydrolysis by human digestive enzymes, allowing polysaccharides to reach the colon relatively intact and exert microbiota–modulating and mucosa–protective effects (125). On the other hand, polysaccharides linked by β-glycosidic bonds can be recognized by intestinal pattern recognition receptors, thereby modulating host immune inflammatory responses. Oat–derived β-glucan, for instance, is recognized by TLR4 and Dectin−1 receptors and exerts protective effects in models of CD (126). A β-1,3–glucan fraction purified from baker's yeast alleviates colonic inflammation by inhibiting the MAPK signaling pathway and reducing the expression of the pro–inflammatory mediators iNOS, IL−6, and IL−1β (127).

The activity of pectic polysaccharides is closely associated with their main–chain structural domain composition. The core structural domains of pectin include the linear homogalacturonan (HG) region and the rhamnogalacturonan–I (RG–I) region, which carries abundant neutral sugar side chains. The arabinan and galactan side chains of the RG–I region are considered the core structures responsible for its biological activity (122). Several comparative studies support this conclusion. Sun et al. compared the anti–colitis activities of okra pectin (OP), citrus pectin (CP), apple pectin (AP), and hawthorn pectin (HP), and found that OP exhibited the highest RG–I region abundance and showed the strongest anti–inflammatory effects in terms of inhibiting the JAK/STAT signaling pathway and activating the Nrf2/Keap1 pathway, whereas AP and HP, which are rich in the HG domain, displayed relatively weak activity (120). Wu et al. further demonstrated a structure–activity relationship in terms of domain composition: pomelo pectin, which is dominated by highly esterified HG, showed only weak anti–nflammatory effects; dragon fruit pectin (RG–I backbone), lemon pectin (arabinan–rich side chains), and potato pectin (galactan–rich side chains) all exhibited moderate IBD–protective activity; in contrast, goji berry pectin and raspberry pectin, which possess a balanced domain composition comprising HG, RG–I, arabinan, and galactan, showed the strongest anti-IBD activity among the tested samples, achieving multifaceted effects including inflammation suppression, intestinal barrier repair, gut microbiota modulation, and antioxidant activity (122).

#### 4.4.4 Side-chain modifications and substituent groups

The biological activity of polysaccharides is determined not only by their main-chain structure but also by the chemical groups present on their side chains. Introducing functional groups into polysaccharide chains is a common modification strategy that can directly alter their physicochemical properties, thereby enhancing biological activity or even conferring new functional characteristics (128). Among the various modification approaches, phosphorylation is primarily employed to reduce viscosity and improve water solubility (129). In contrast, sulfation has been shown to exert more pronounced effects on immunomodulatory activity. For instance, among four polysaccharide fractions isolated from the edible green alga Caulerpa lentillifera, CLGP4, which contained the highest sulfate content (21.26%), exhibited the strongest anti-inflammatory activity in LPS-stimulated HT29 cells, significantly inhibiting IL-1β and TNF-α production and altering the secretion of SIgA and MUC2 (130). Similarly, sulfated Chinese yam polysaccharide (S-CYP) has been shown to regulate the MAPK signaling pathway and enhance macrophage immune responsiveness in a co-culture model, partly through increased secretion of IL-1β and TNF-α (131). In addition to phosphorylation and sulfation, other modification strategies, including carboxymethylation, acetylation, esterification, thiolation, and glycosylation, can also enhance the biological activity of polysaccharides to varying degrees (132).

It should be noted, however, that structure–activity relationships are not governed by simple linear correlations. The same structural feature may exert opposite effects in polysaccharides from different sources, and structure–activity relationships observed *in vitro* do not always align with those observed *in vivo*. Currently, most studies report the activity of individual polysaccharides without systematic comparisons of key structural parameters. To provide an intuitive overview of the structural and functional characteristics of polysaccharides from different sources, we have compiled Supplementary Table S1, which summarizes the sources, key structural features, and major IBD-related activities of representative dietary polysaccharides.

## 5 Conclusion and outlook

### 5.1 Summary of key findings

With the worldwide prevalence of IBD continuing to rise and the currently available pharmacological treatments being limited by side effects and incomplete efficacy, there is an urgent need to develop new therapeutic strategies. Accumulating evidence indicates that dietary polysaccharides play a critical role in the prevention and management of IBD (9, 78, 117, 122, 133).

**Figure 2:**
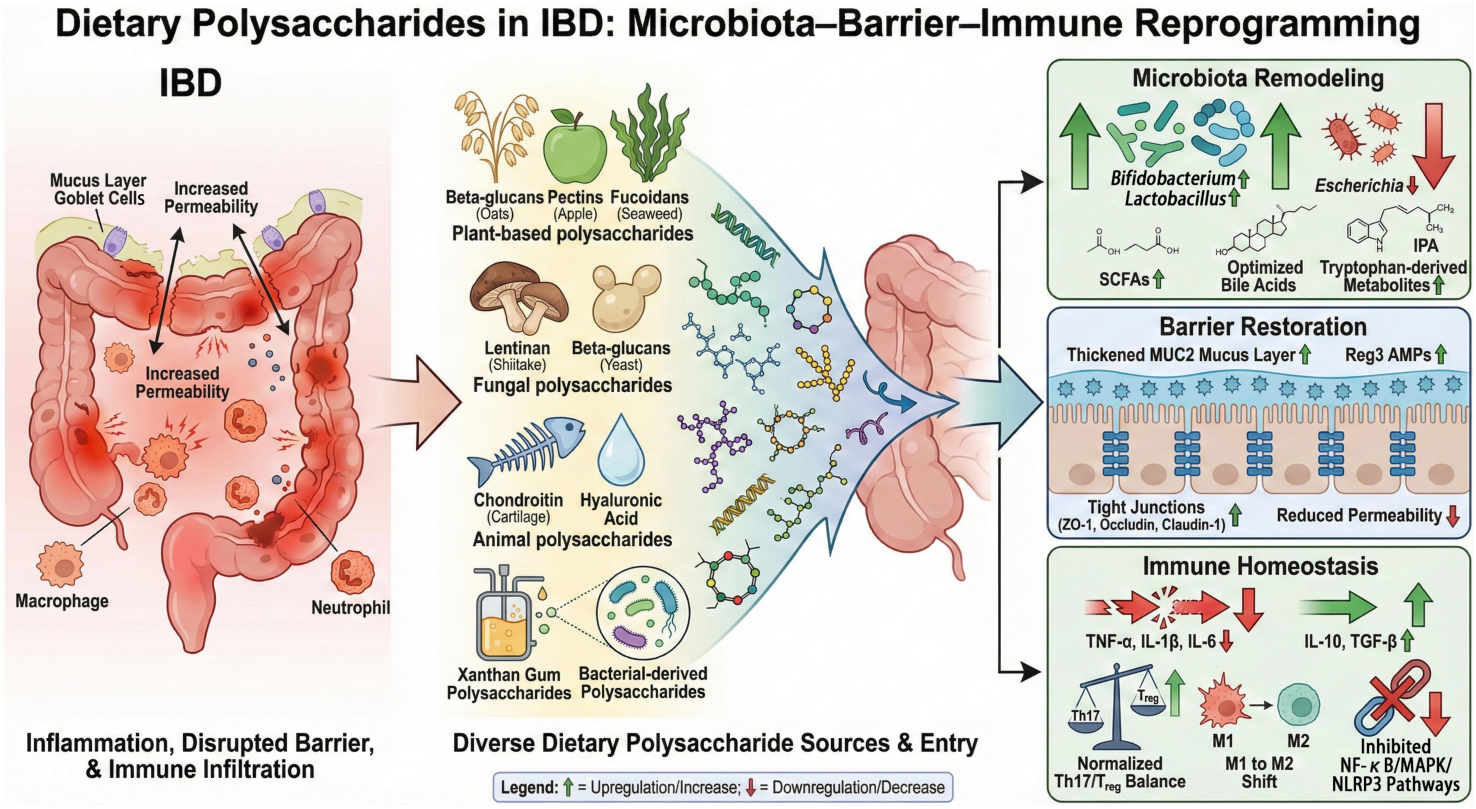
Mechanistic Studies on the Treatment of IBD with Dietary-Derived Polysaccharides. In IBD, intestinal inflammation is characterized by barrier disruption, increased permeability, and immune cell infiltration. Dietary polysaccharides derived from plant (e.g., pectins, β-glucans, fucoidans), fungal (e.g., lentinan), animal (e.g., chondroitin, hyaluronic acid), and microbial sources (e.g., xanthan gum) reach the colon and serve as fermentable substrates for the gut microbiota. Through microbial fermentation and metabolic transformation, these polysaccharides promote microbiota remodeling, characterized by enrichment of beneficial bacteria (e.g., *Bifidobacterium, Lactobacillus*) and suppression of pathobionts (e.g., *Escherichia*), accompanied by increased production of bioactive metabolites such as short-chain fatty acids (SCFAs), optimized bile acids, and tryptophan-derived metabolites. These changes contribute to restoration of the intestinal barrier by enhancing MUC2 mucus layer thickness, upregulating tight junction proteins (ZO-1, occludin, claudin-1), and reducing epithelial permeability. Simultaneously, polysaccharides modulate immune homeostasis by suppressing pro-inflammatory cytokines (TNF-α, IL-1β, IL-6), promoting anti-inflammatory responses (IL-10, TGF-β), facilitating macrophage polarization from M1 to M2, and inhibiting NF-κB/MAPK/NLRP3 signaling pathways. Together, these effects illustrate a coordinated microbiota–barrier–immune regulatory axis underlying the therapeutic potential of dietary polysaccharides in IBD.

In this review, we summarized the pathogenic mechanisms of IBD and the biological activities of dietary polysaccharides, and highlighted their marked beneficial effects in attenuating colonic inflammation. The protective actions of these polysaccharides involve multiple, interrelated mechanisms. They modulate the gut microbiota by restoring microbial ecology and regulating microbiota-derived metabolites, repair both mechanical and chemical components of the intestinal barrier, and reshape cytokine networks and immune cell responses while targeting key inflammatory signaling pathways. Together, these processes contribute to the suppression of aberrant inflammatory responses in the gut (Figure 2).

### 5.2 Challenges in clinical translation of dietary polysaccharides in IBD

Despite substantial progress in preclinical research, current evidence supporting the efficacy of dietary polysaccharides in IBD is still derived predominantly from animal studies and *in vitro* or *ex vivo* experiments, and translation into clinical practice remains challenging. One major obstacle is standardization and quality control, as the structural characteristics of polysaccharides, including molecular weight and monosaccharide composition, are highly influenced by raw material sources and extraction procedures. This variability may result in batch-to-batch differences in bioactivity and complicate the development of reproducible preparations. In addition, the bioavailability and *in vivo* metabolic fate of dietary polysaccharides remain insufficiently understood, and the mechanisms underlying their degradation, absorption, and targeted delivery after oral administration require further clarification. Another critical challenge is patient heterogeneity. Given the substantial differences in genetic background, gut microbiota composition, and disease subtype among patients with IBD, the same polysaccharide may produce variable therapeutic responses across individuals. More importantly, the current lack of high-quality clinical evidence substantially limits the incorporation of dietary polysaccharides into evidence-based clinical recommendations. Overall, the structure–activity relationships, *in vivo* metabolic behavior, and clinical translation pathways of dietary polysaccharides require further systematic investigation.

### 5.3 Future perspectives

Future research should focus on elucidating how polysaccharides with different dietary origins, structures, and molecular weights differentially influence immune cells, intestinal epithelial cells, and the gut microbiota. Integration of emerging technologies, including nanotechnology and artificial intelligence, will be valuable for clarifying their mechanisms of action and for guiding the rational design of polysaccharide-based therapeutics, dietary supplements, and functional foods tailored to the individual characteristics of patients with IBD. However, the translational potential of these technologies still requires validation in rigorous clinical settings, and their current applicability remains limited by challenges such as data standardization, model interpretability, and *in vivo* safety evaluation.
