# Supplementary material for: Dietary polysaccharides in the management of inflammatory bowel disease: recent advances
Source: Front Nutr. 2026 May 11;13:1779617. doi: 10.3389/fnut.2026.1779617 (PMC13199799; doi:10.3389/fnut.2026.1779617)
Supplement: Supplementary file 1 [file Table_1.DOCX]

**Supplementary** **Table 1. Representative dietary polysaccharides with different structural features and their major biological effects in IBD-related models**

| Structural feature | Representative polysaccharides | Dominant biological outcomes |
| --- | --- | --- |
| Relatively low to medium molecular weight | Low-molecular-weight Astragalus polysaccharide; low-molecular-weight blackberry polysaccharide; citrus pectin (CP); okra pectin (OP); low-molecular-weight *Tremella fuciformis* polysaccharide (TFLP) | Enhanced water solubility and intestinal bioavailability; promotion of short-chain fatty acid (SCFA) production; inhibition of the NF-κB signaling pathway; reduction of ubiquitin-mediated degradation of ZO-1; stabilization of the intestinal mechanical barrier; prebiotic and anti-inflammatory effects |
| β‑Glucan backbone | Oat β-glucan; baker’s yeast β-1,3-glucan | Resistance to hydrolysis by human digestive enzymes; recognition by TLR4 and Dectin-1 receptors; inhibition of the MAPK signaling pathway; downregulation of iNOS, IL-6, and IL-1β expression |
| Arabinose-rich / arabinogalactan-related structures | Lemon pectin; banana arabinogalactan; goji berry arabinogalactan (LBP-m) | Inhibition of IL-6, TNF-α, and IL-1β; promotion of IL-10 production; regulation of the NF-κB/MAPK/PPARγ signaling pathways and NLRP3 inflammasome activity; modulation of gut microbiota and butyrate metabolism; activation of the Nrf2/HO-1 pathway; upregulation of tight junction protein expression |
| RG-I-rich domain | Okra pectin (OP); dragon fruit pectin; goji berry pectin; raspberry pectin | Inhibition of the JAK/STAT signaling pathway; activation of the Nrf2/Keap1 pathway |
| Sulfated modification | *Caulerpa lentillifera* polysaccharide CLGP4; sulfated Chinese yam polysaccharide (S-CYP) | Modulation of inflammatory cytokine secretion; regulation of the MAPK signaling pathway; alteration of mucosal immune-related factors; influence on macrophage immune responsiveness |
